# Profiling the HER3/PI3K Pathway in Breast Tumors Using Proximity-Directed Assays Identifies Correlations between Protein Complexes and Phosphoproteins

**DOI:** 10.1371/journal.pone.0016443

**Published:** 2011-01-28

**Authors:** Ali Mukherjee, Youssouf Badal, Xuan-Thao Nguyen, Johanna Miller, Ahmed Chenna, Hasan Tahir, Alicia Newton, Gordon Parry, Stephen Williams

**Affiliations:** Department of Oncology, Monogram Biosciences, South San Francisco, California, United States of America; Istituto Dermopatico dell'Immacolata, Italy

## Abstract

**Background:**

The identification of patients for targeted antineoplastic therapies requires accurate measurement of therapeutic targets and associated signaling complexes. HER3 signaling through heterodimerization is an important growth-promoting mechanism in several tumor types and may be a principal resistance mechanism by which EGFR and HER2 expressing tumors elude targeted therapies. Current methods that can study these interactions are inadequate for formalin-fixed, paraffin-embedded (FFPE) tumor samples.

**Methodology and Principal Findings:**

Herein, we describe a panel of proximity-directed assays capable of measuring protein-interactions and phosphorylation in FFPE samples in the HER3/PI3K/Akt pathway and examine the capability of these assays to inform on the functional state of the pathway. We used FFPE breast cancer cell line and tumor models for this study. In breast cancer cell lines we observe both ligand-dependent and independent activation of the pathway and strong correlations between measured activation of key analytes. When selected cell lines are treated with HER2 inhibitors, we not only observe the expected molecular effects based on mechanism of action knowledge, but also novel effects of HER2 inhibition on key targets in the HER receptor pathway. Significantly, in a xenograft model of delayed tumor fixation, HER3 phosphorylation is unstable, while alternate measures of pathway activation, such as formation of the HER3PI3K complex is preserved. Measurements in breast tumor samples showed correlations between HER3 phosphorylation and receptor interactions, obviating the need to use phosphorylation as a surrogate for HER3 activation.

**Significance:**

This assay system is capable of quantitatively measuring therapeutically relevant responses and enables molecular profiling of receptor networks in both preclinical and tumor models.

## Introduction

A goal of modern molecular cancer diagnostics is to identify the underlying molecular signature of cancers on a patient-by-patient basis to guide the selection of an appropriate therapeutic regimen [1]. The capability to measure individual proteins, protein trafficking and localization, protein-protein interactions and protein phosphorylation are key requisites to deduce pathway activation and correlate specific signaling events with biological outcomes such as cell growth and survival or resistance/sensitivity to therapeutic treatments [2]. Obtaining such measurements from formalin-fixed, paraffin-embedded (FFPE) samples is necessary since patient biopsies are routinely preserved in this format for histological assessment: unfortunately, biochemical techniques suitable for this sample type are severely limited. Measurement of protein expression and protein phosphorylation by immunohistochemistry is valuable, but reveals only a partial picture of the signaling network [3] and is limited by the availability of phosphorylation site-specific antibodies and lability of certain phosphorylation sites reviewed in [4]. The measurement of protein-protein interactions is routine from cell or tissue lysates using standard techniques such as co-immunoprecipitation and Western blotting, but few techniques are available for FFPE samples. Besides the approach described in this publication, to our knowledge the only other assays capable of measuring protein complexes in FFPE tissue sections are Fluorescence Resonance Energy Transfer [5] and in situ proximity ligation assay [6], [7].

The ErbB family of receptor tyrosine kinases (RTKs) is essential for normal cellular development [8]–[10]; however, several proteins that mediate ErbB signaling contribute to tumorigenesis in rodents and humans [11], [12]. The ErbB family is comprised of four members: EGFR/ErbB1/HER1, ErbB2/NeuHER2/, ErbB3/HER3 and ErbB4/HER4 [9]. Both ligand-induced and ligand-independent dimerization and activation of HER receptors are known to occur [9], including formation of the HER2-HER3 heterodimer in HER2 amplified cells [13]. Dimerization is followed by receptor transactivation and phosphorylation, the recruitment of various cytosolic proteins to the phosphorylated receptors, thereby triggering various signaling cascades including the PI3K/Akt, PKC, MAPK and the Ras signaling pathways [14]–[18].

The measurement of biomarker expression levels has been successfully employed for selecting patients for monoclonal antibody-based targeted therapy, as in the treatment with trastuzumab (anti-HER2 humanized antibody) for HER2 overexpressing breast cancer [19], [20]. However, even the measurement of HER2 expression levels has low positive predictive value: the objective response rate for patients selected for trastuzumab therapy is less than 35% [21], [22]. Utilization of alternate signaling pathways, in particular, heterodimerization of HER family members, is often responsible for de novo and acquired resistance to HER-targeted therapies [22], [23]. Dimerization of HER3 with HER2 is known to be one of the most mitogenic protein complexes [24] and is frequently implicated as a potential drug resistance mechanism [25], [26]. Conversely, HER3 has also been identified as a potential marker of drug sensitivity. A phase II study of lapatinib in HER2-positive inflammatory breast cancer showed that, phosphorylated HER3 predicted response to lapatinib and tumors coexpressing phosphorylated HER2 and HER3P were more likely to respond [27]. As a prognostic marker, expression of HER3 has been associated with reduced survival in melanoma, gastric and breast cancer [28], [29], although a more complete picture of the role of HER3 in this regard is yet to emerge. As an addition to measuring the total HER3 protein, the capability to measure HER3-phosphatidylinositol-3 kinase (HER3PI3K) pathway activation could enhance the predictive and prognostic value of measurement in this pathway. The ability to measure HER3 activation has become more critical as HER3-targeted therapies are showing promising results in pre-clinical studies [30].

A previous study demonstrating the use of VeraTag™ technology in proteomics research reported on the development of receptor expression and homodimerization assays [31]. In this report, we describe the development of a panel of proximity-directed assays to measure protein interactions and protein phosphorylation in the HER3PI3K pathway using FFPE samples. We investigate the utility of these assays in measuring clinical responses in breast cancer cell-lines and in the molecular characterization of human breast cancer tumors. In summary, our findings demonstrate a number of viable assays measuring HER3-complex formation that are useful for assessing HER3 activity in patient tumor samples.

## Materials and Methods

### Ethics statement

Ethics committee approval of the study was not sought since no human or animal experiment was performed by the authors. Mouse tumor xenografts and human tumor FFPE blocks were purchased from commercial vendors (Charles River Laboratories and Asterand respectively). The humane care and use of laboratory animal policy of CRL can be found at http://info.criver.com/about_charles_river/humane_care_initiative/index.html. The bioethics and privacy policy for Asterand can be found at http://www.asterand.com/Asterand/about/ethics.htm.

### VeraTag assays

Cancer cell-lines and tumors were analyzed in VeraTag assays to determine HER2-HER3 heterodimers (HER23D), pan HER3 phosphorylation (HER3P), HER3PI3K, Akt serine 473 phosphorylation (pAkt) and total Akt (tAkt).

### Cell culture and treatment

All cell-lines were purchased from ATCC. Cells were grown to confluence and were serum starved overnight prior to heregulin (HRG) stimulation. Cells were treated with either 200 nM 2C4 (murine pertuzumab, ATCC), 200 nM trastuzumab (Myoderm), 1 µM lapatinib (LC Laboratories) or left untreated for 2 h. HRG stimulation (60 nM) was done after drug treatment prior to lysis or fixation and FFPE block preparation. Non-stimulated cells were lysed or fixed directly after drug treatment.

### FFPE assays

**Figure 1:**
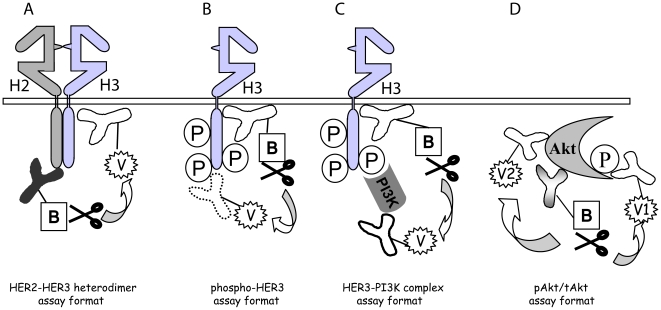
Schematic representation of the VeraTag assays. A pair of antibodies, one of which is conjugated to biotin (B) and the other a fluorescent molecule suitable for analysis by CE (VeraTag; V), bind to distinct epitopes on a protein or a protein complex. The VeraTag molecules are attached to the antibodies via photo-cleavable linkers. Each target measured in the assay is assigned to a unique VeraTag. Methylene blue, conjugated to streptavidin, binds to the biotin-labeled antibody and is photo-activated by red light. The released singlet oxygen, as a result of methylene blue catalyzed photosensitization, cleaves VeraTag molecules in close proximity to the antibody-biotin-streptavidin complex. (A) HER2-HER3 heterodimers are measured using HER2- and HER3-specific antibodies. (B) Total HER3 phosphorylation is measured using a HER3-specific and a generic phosphotyrosine antibody. (C) Interaction of PI3K and HER3 is measured using a HER3-specific antibody and an antibody directed to the p85 component of the PI3K protein. (D) Two distinct VeraTag molecules that can be separated by CE are used in the Akt assay, one for pAkt and the other for tAkt. Total- and phospho-Akt are measured using a pair of antibodies directed to Akt and a third VeraTag labeled antibody to a specific phospho-site on Akt.

FFPE blocks, antibody conjugates and molecular scissors were prepared for the analyses as detailed in supporting information (Text S1) and in previous reports [31], [32]. The principle of the VeraTag assay is described in Figure 1. The general assay protocol is represented by the schematic in Figure S1. The workflow of the assay is depicted in Figure S2. Antigen retrieval was performed on the dewaxed and hydrated sections on positively charged glass slides in Tris buffer (DAKO) in a pressure cooker (Biocare Medical) following their protocols. Assays were performed with the following antibodies: HER3P, biotinylated HER3 antibody (mouse monoclonal, B9A11; Monogram) and VeraTag-labeled generic p-tyrosine antibody (PT100; CST), HER3PI3K, biotin-labeled B9A11 and a VeraTag-labeled antibody to the alpha isoform of the p85 subunit (05-212; Millipore), HER23D, biotin-labeled B9A11 and VeraTag-labeled HER2 antibody (Ab-8; Labvision). For the tAkt-pAkt duplex assay, a specific VeraTag labeled pAkt antibody (4051; CST) was used in combination with a biotin-labeled Akt1 antibody (SC-1619; Santa Cruz) and a VeraTag labeled Akt antibody (SC-5298; Santa Cruz). For the paired isotype control (ITC) experiments, the biotin-labeled antibody was replaced by a biotin-labeled mouse IgG_1_ (BD Biosciences), while all other assay conditions remained the same. After antibody incubation, streptavidin-methylene blue (SA-MB) was added to the tissue section followed by photo-cleavage to release VeraTags, which were separated by capillary electrophoresis (CE) and quantitated. The details of assay characterization are provided in Text S1. In general each assay has a high level of specificity and precision (Figure S3, Figure S4 and Figure S5).

### Generation and preparation of N87 xenografts

N87 xenografts (Charles River Laboratories) were generated following an established protocol. Briefly, 30 mg to 70 mg tumor fragments from N87 xenografts were implanted into nude mice (3 per group). Tumors were excised from each animal and half of the tumor was placed into fixative for block-making. Tumor powders prepared from the remaining half were placed in liquid nitrogen. The powders were thawed in lysis buffer [1% Triton X-100, 50 mM Tris-HCl (pH 7.5), 35 mM NaCl, 50 mM NaF, 50 mM sodium beta-glycerophosphate, 1 mM Na_3_VO_4_, 5 mM EDTA and 1 tablet (per 10 ml) complete protease inhibitor (Roche) in de-ionized water] before protein estimation and SDS-PAGE. Tumors from mice of group 1 (G1) were frozen or fixed immediately. Mice from group 2 (G2) were prepared such that 15 min elapsed from harvest to processing while remaining at room temperature. Mice from group 3 (G3) were prepared with 30 min elapsing, also at room temperature until processing. Mice from group 4 (G4) were prepared with 30 min elapsing but at 4°C. Mice from group 5 (G5) were prepared with 120 min elapsing at room temperature.

### SDS-PAGE and Western blot analysis

For HER3 immunoprecipitation, rabbit polyclonal antibody SC-285 (Santa Cruz) was incubated with protein G beads (Pierce) in 100 mM HEPES (pH 8.2), 150 mM NaCl buffer at 4°C for 2 h. The beads were washed and 300 µg of lysate protein was immunoprecipitated overnight at 4°C with 20 µL antibody-protein G beads. After immunoprecipitation, the beads were washed twice with lysis buffer. SDS-sample buffer containing an additional 50 mM dithiothreitol was added to each precipitate, and the samples were boiled for 4 min. The samples were run on a 4–12% bis-tris gel and transferred to a PVDF membrane. Co-immunoprecipitating HER2, p85 and phosphoproteins were analyzed by Western blot with Ab-8, 05-212 or 4G10 antibodies respectively. Western blotting for Akt and phospho-Akt proteins was performed using SC-5298 (Santa Cruz) and 4051 (CST) antibodies respectively.

### Estimation of tumor area

All slides underwent haematoxylin and eosin (H&E) staining after the completion of VeraTag assays followed by estimation of the tumor area by a certified pathologist. To the extent possible, tumor area measurement was restricted to regions of invasive carcinoma only. Samples with significant necrosis, insignificant tumor content or ductal carcinoma in situ were excluded from analysis.

### Statistical Analysis

Pearson correlation coefficients (r) were used to determine correlations between the measured targets and a two-tailed p-value less than 0.03 was considered to represent a statistically significant correlation. The significance of assay measurements between untreated and treated samples was assessed by Student's t test. Error bars denote the SEM for three measurements unless noted otherwise.

## Results

### Measurement of ligand-induced changes in HER23D, HER3P, HER3PI3K, tAkt and pAkt in cell-lines in FFPE format

**Figure 2:**
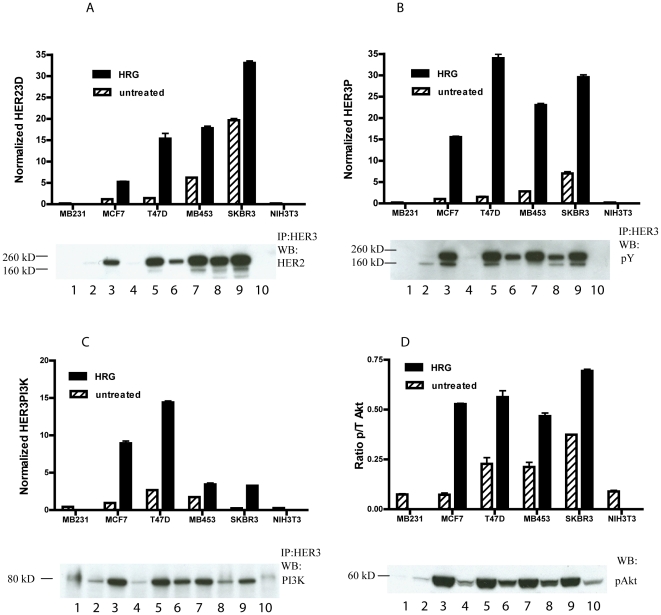
HER2-HER3 heterodimer, phospho-HER3, HER3-PI3K, phospho-Akt analysis of breast cancer cell-lines. The indicated breast cancer cell-lines with and without HRG stimulation were used in VeraTag FFPE assays. Data from HER23D (A), HER3P (B), HER3PI3K (C) and Akt (D) FFPE assays were analyzed and the results are plotted in the graphs and the corresponding co-IP WB (A–C) and pAkt western (D) were performed on the same cell lysate for comparison. The normalized RPA values are plotted on the y-axis. pAkt data is plotted as a function of tAkt. HRG-stimulated signal is denoted by the solid bars and unstimulated signal by hatch-marked bars. Western lanes are as follows: 1- MB231, 2-MCF7, 3-MCF7_H, 4-T47D, 5-T47D_H, 6-MB453, 7-MB453_H, 8-SKBR3, 9-SKBR3_H, 10- NIH3T3. Underscore H (_H) indicates cells were treated with HRG.

Figures 1A–D depict the FFPE assays for heterodimer, phosphoprotein and protein-complex measurements used in this study. A panel of breast cancer cell-lines was selected for coexpression of a range of HER2 and HER3 receptors (Table S1). As expected, HRG strongly activated the HER3/PI3K/Akt pathway in these breast cancer cell-lines [33]. Ligand stimulation resulted in a 5-fold increase in HER23D, 15-fold increase in HER3P, 10-fold increase in HER3PI3K complex and 5-fold increase in the ratio of phospho-to-total Akt (pAkt/tAkt) levels in MCF7 cells (Figures 2A–D). The total HER2, HER3 and Akt protein levels remain relatively unchanged (Figure S6). The cells with highest levels of HER2 had high basal levels of HER23D (SKBR3), whereas the low HER2 cell-lines (T47D, MCF7) had low basal HER23D and showed the highest fold-change inductions with HRG. The ligand-independent dimer is an active species consistent with the phosphorylation of the downstream targets measured (HER3P/pAkt). The ligand-independent HER23D and HER2T signals as measured by the VeraTag FFPE assays are well correlated and the same pattern is observed in data from VeraTag lysate assays (Figure S7).

As part of the cross-validation of the VeraTag assay system, we compared results obtained from FFPE assays to those from conventional formats like co-immunoprecipitation followed by SDS-PAGE and Western blotting. Figure 2 shows receptor complexation and phosphorylation by co-immunoprecipitation/Western blotting in the same panel of breast cancer cell-lines used in the VeraTag FFPE assays and there is generally good correlation between the cell lysate and FFPE measurements. In cell-lines with high HER2 expression the ligand-independent dimers constitute a significant proportion of the dimer signal. Additionally, in high HER2 cell lines HRG expression is low [34] suggesting that the presence of HER23 heterodimers is ligand-independent.

### VeraTag assay in breast tumor samples

**Figure 3:**
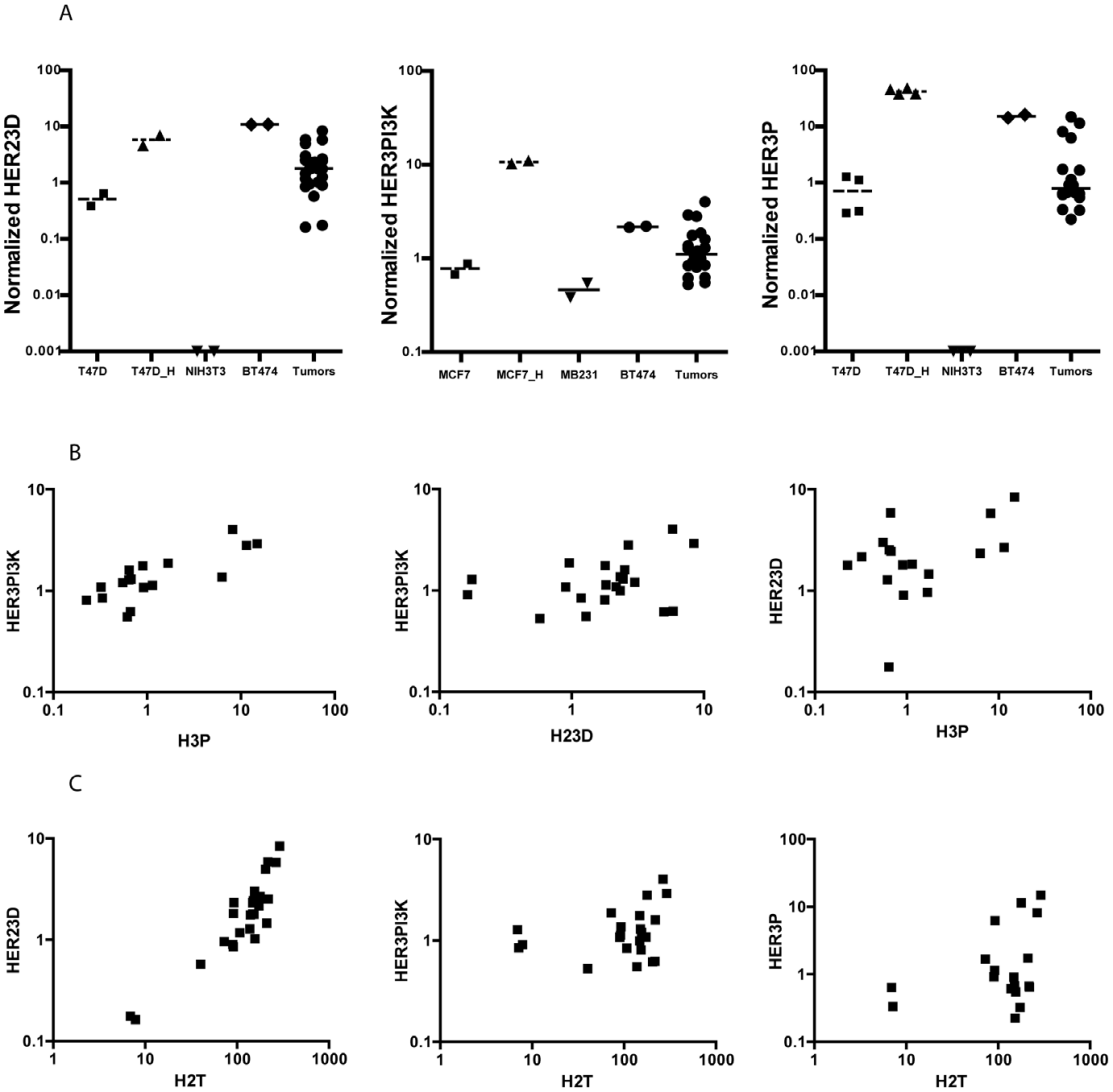
VeraTag assays to monitor signaling pathways in breast tumors. FFPE sections from HER2-positive tumors were subjected to HER23D, HER3P and HER3PI3K VeraTag assays (A). Cell-line controls for each assay were used as indicated on the x-axis. Underscore H (_H) indicates cells were treated with HRG. The normalized RPA values after correction for ITC signal was plotted on the y-axis. (B) Correlations between HER23D, HER3P and HER3PI3K signal from VeraTag assays. (C) Correlations between HER2T and markers of HER3 activation (HER23D, HER3P and HER3PI3K) from VeraTag assays.

Activation of the HER3/PI3K/Akt pathway in breast tumors has often been implicated in resistance to HER2-targeted therapies. We screened a set of 27 breast tumors to determine our ability to measure HER23D, HER3P and HER3PI3K in tumors and to ascertain the utility of such measurements in a clinical setting (Figure 3A). We used cell-line controls to span the dynamic range of the measurements, allowing for normalization of the data across multiple batches and ensuring our ability to compare different batches. The HER23D measurement showed a dynamic range of greater than 2 logs, while both the HER3P and the HER3PI3K assays had dynamic range of 1.5 logs. We excluded from analysis tumors that showed specific signal less than two-fold of the ITC background (negative). By this criterion, 22 of the tumors were deemed positive by VeraTag HER3PI3K assay, while 18 of these tumors showed HER3 activation as measured by phosphorylation of HER3.

### Correlations between protein expression, dimerization and phosphorylation levels

As observed in cell-lines, the HER23D levels track strongly with the levels of HER2 in these tumors (p-value<0.0001). Additionally, the HER3P assay shows a statistically significant association with HER23D (correlation coefficient r of 0.65, p-value  = 0.0026) and the HER3PI3K measurement trends with HER23D measurements (r = 0.36), although does not reach statistical significance in this set of tumors. HER3PI3K and HER3P measurements (Figure 3B) show good correlation (r = 0.57, p-value = 0.01). HER2T measurements (Figure 3C) also co-vary with both HER3P and HER3PI3K measurements (p-value<0.03). There is a trend for association between HER3T and HER23D measurements (p-value = 0.078) but not between HER3T and HER3PI3K, or HER3T and HER2T (p-values>0.15). These correlations are presented in Table 1.

**Table 1:** Correlations of HER3/PI3K pathway analytes in breast tumors.

Correlations	n#	r^2^	**p-value
HER2T and HER3T	27	.065	.1993
HER2T and HER23D	25	.670	**<0.0001**
HER2T and HER3P	18	.254	.0329
HER2T and HER3PI3K	22	.266	**.0140**
HER3T and HER23D	25	.129	.0776
HER3T and HER3P	18	.093	.2195
HER3T and HER3PI3K	22	.158	.0671
HER23D and HER3P	17	.445	**.0035**
HER23D and HER3PI3K	21	.246	**.0222**
HER3P and HER3PI3K	17	.617	**.0002**

*The normalized RPA values after correction for ITC signal was used in the analysis.

# The number of tumors (n) eligible for each analysis.

§ The correlation coefficients (Pearson r) were calculated and expressed as r^2^.

**Two-tailed p-value was determined and the correlation was considered statistically significant when p<0.03, shown in bold.

### Modulation of the HER2/HER3 pathway with targeted therapies

**Figure 4:**
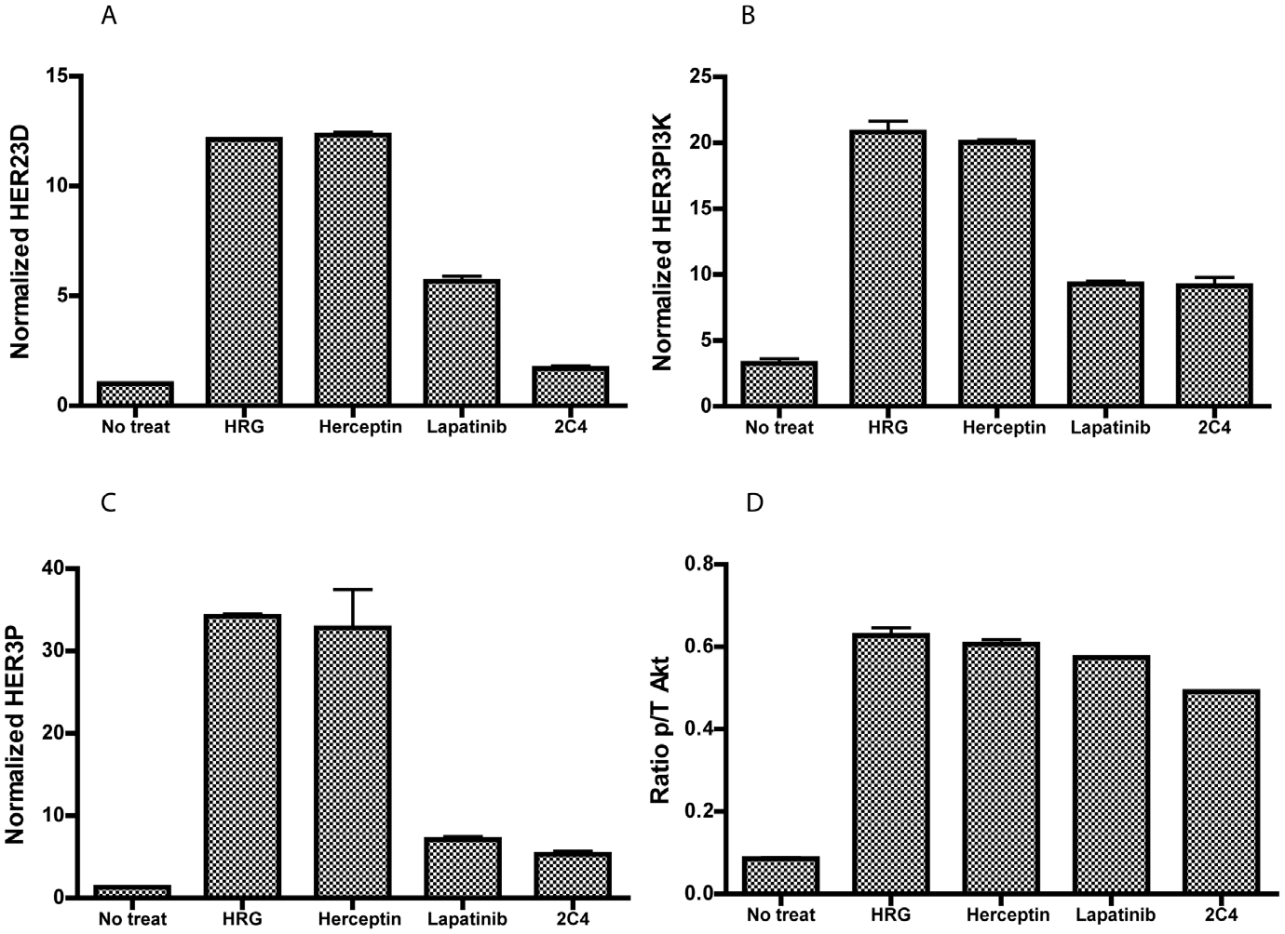
Effects of trastuzumab, 2C4 and lapatinib on molecular markers under HRG-stimulated conditions. T47D cells were exposed to trastuzumab, 2C4, lapatinib or left untreated prior to treatment with heregulin. VeraTag assays to measure (A) HER23D, (B) HER3P, (C) p/t-Akt and (D) HER3PI3K were performed on FFPE sections prepared from these cells. The normalized RPA values are plotted on the y-axis. The treatments are indicated on the x-axis.

In order to measure the inhibition of HER2 mediated HER3 activation, we tested three drugs that target HER2 (2C4, trastuzumab and lapatinib) using FFPE cell-lines as our model system. When low HER2-expressing T47D cells are exposed to the inhibitors followed by ligand stimulation, relative to non-drug treated control, HER23D is downregulated by 2C4 (∼7-fold, p-value = 0.00014) and to a lesser extent by lapatinib, but not by trastuzumab (Figure 4). Trastuzumab is unable to block the ligand-induced increases in HER3P, HER3PI3K and pAkt/tAkt either. Lapatinib downregulated HER3P (∼6-fold) and HER3PI3K, in a manner similar to 2C4. The inhibitors had negligible effect on pAkt/tAkt in HRG-stimulated T47D cells. The levels of total HER2, HER3, PI3K, and Akt are relatively unchanged (data not shown).

**Figure 5:**
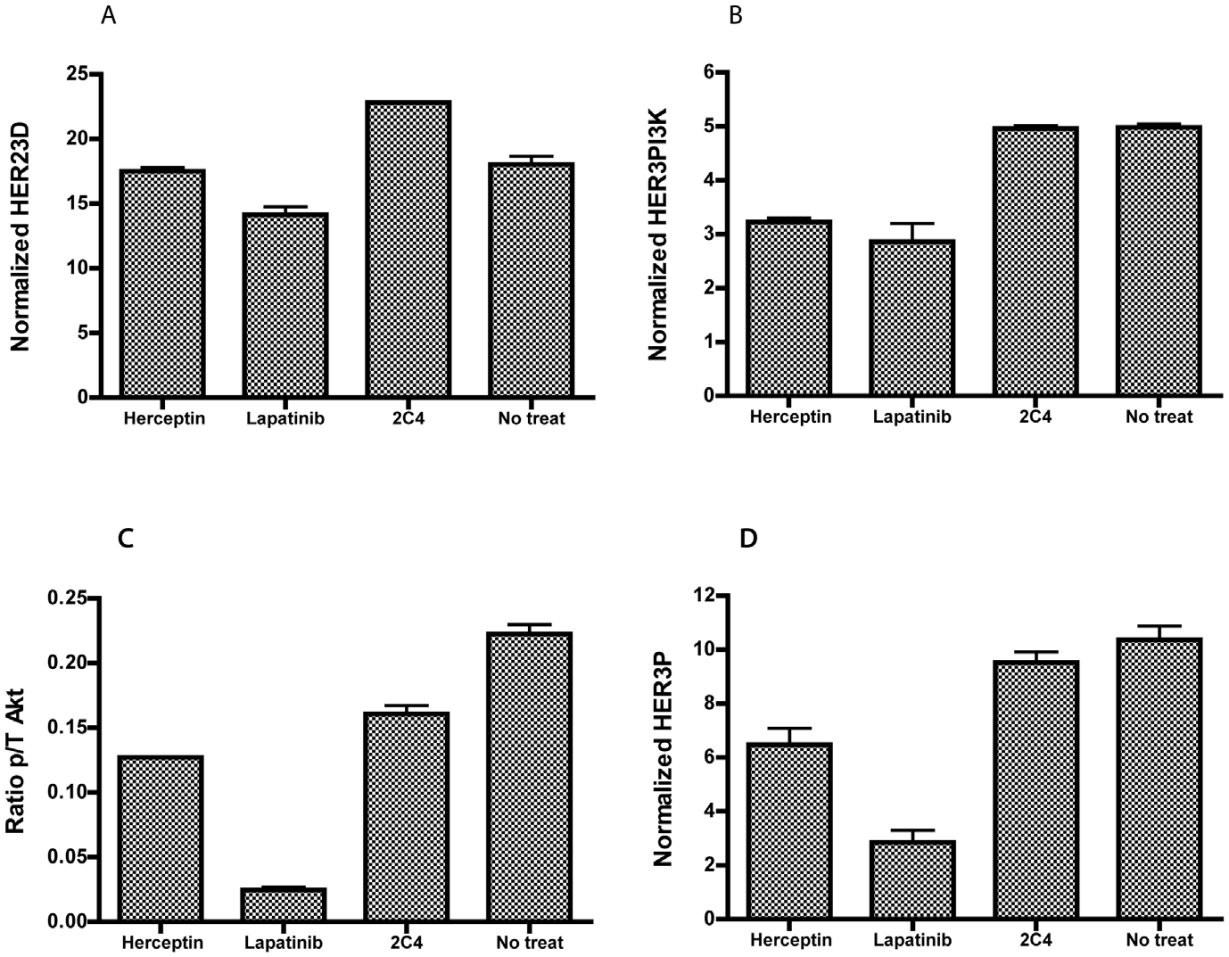
Effects of trastuzumab, 2C4 and lapatinib on molecular markers of basally activated breast cancer cells. BT474 cells were exposed to trastuzumab, 2C4, lapatinib or left untreated. VeraTag assays to measure (A) HER23D, (B) HER3P, (C) p/t-Akt and (D) HER3PI3K were performed on FFPE sections prepared from these cells. The normalized RPA values are plotted on the y-axis. The treatments are indicated on the x-axis.

As expected, 2C4 is not able to downregulate HER23D in cells expressing basal levels of this heterodimer, as in BT474 (Figure 5). In fact, 2C4 does not have a significant inhibitory effect on any target measured in BT474 except for a small change in pAkt/tAkt. Trastuzumab, however, blocks basal activation of this pathway as evidenced by the downregulation of both HER3PI3K and pAkt/tAkt in BT474 (p-values <0.006). There is a downward trend on HER3P, but we detected no measurable effect of trastuzumab on HER23D. Lapatinib inhibits all four targets to varying degrees, with the strongest effect (∼5-fold) on pAkt/tAkt (p-value = 0.0014) in BT474. The cross-validation of the FFPE assay data for both the cell-lines is presented in Figure S8 and Figure S9.

### Measurement of HER3 activation in xenografts

**Figure 6:**
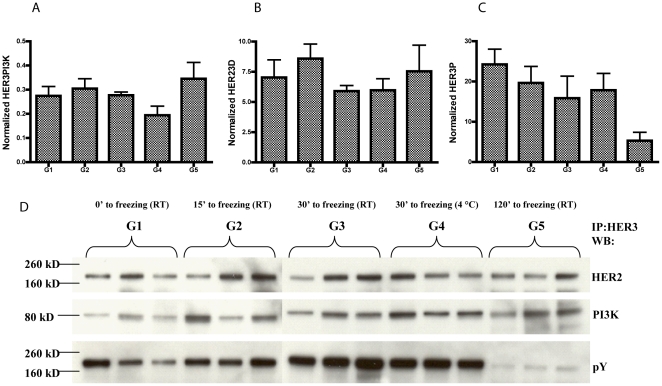
VeraTag assays to monitor signaling pathways in N87 xenograft tumors. HER3PI3K (A), HER23D (B) and HER3P (C) VeraTag assays were performed on tumors obtained from the five groups of mice (G1–G5). The normalized RPA values are plotted on the y-axis. The error bars denote the standard error of the mean (SEM) from measurements of the three mice per time point. Equal amounts of total protein from the tumors of the five groups of mice (three mice per group) were subjected to immunoprecipitation using anti-HER3 antibody (D). SDS-PAGE followed by western blotting was performed to detect HER23D, HER3P and HER3PI3K as described in Material and Methods.

We used the N87 gastric cancer xenograft model and the suite of HER3-related VeraTag assays to examine the applicability of these assays in tumor models. We chose N87 since this cell line shows detectable levels of both HER2 and HER3 and is known to be HER2-driven [35]. Tumor fragments from N87 were implanted into mice to generate the xenografts. We monitored total protein (Figure S10), protein-complexes and phosphoproteins (Figure 6) using VeraTag FFPE assays.

### Relative stabilities of protein-complexes and phosphoproteins

A major consideration in using solid tumor patient samples for the detection and measurement of prognostic or predictive biomarkers is the stability of the marker pre- and post-fixation. In particular, protein phosphorylation is sensitive to the time between excision of the patient's tumor and fixation in formalin. To investigate the stability of selected targets and the potential loss of receptor phosphorylation due to delayed fixation in a model system, we left the excised N87 xenografts at room temperature for different times up to 2 h post-surgery, before either fixing them in NBF for making FFPE blocks or preparing lysate samples. We performed the HER23D, HER3P and HER3PI3K assays on these samples and found no significant degradation of either HER23D or HER3PI3K at any measured time point up to 2 h of delayed fixation. However, we saw evidence of the degradation of HER3P after 2 h (Figure 6), while total protein remained constant (Figure S10A–B). There is no statistically significant difference in either the HER23D or the HER3PI3K measurements between any of the five groups of mice (p-values >0.5 and >0.2 respectively). The HER3P measurements, however, drop measurably for groups three, four and five. The difference in the HER3P measurements of group one and group five reached statistical significance (p-value = 0.012). As with cell-line data, the data from the FFPE assays correlated well with co-immunoprecipitation/Western Blot from the same tumors.

## Discussion

Despite the success of targeted therapies for the treatment of solid tumors, drug resistance is still a major issue and concern. While many different targeted therapy resistance mechanisms have been advanced based on in vitro cell line models, there has been very little study to ascertain whether these mechanisms are significant in the more complex biological setting of a patient's tumor. What are clearly lacking at this present time are appropriate techniques for the robust examination of activated signaling pathways in FFPE tumor sections. Approaches to accurately and reproducibly measure multiple nodes in a pathway should allow identification of a tumor's oncogenic pathway addiction as well as highlight potential activated resistance pathways that may result in poor, or limited, response to therapy.

We have developed a technology platform that permits quantitative measurements of proteins, protein complexes and phosphorylation in FFPE tumors. In this study, we described the use of a dual antibody-based, proximity-driven assay for the detection and quantitation of receptor dimers and protein complexes in the HER3/PI3K/Akt pathway. Our results indicate we can measure molecular perturbations at key nodes along this pathway either as a function of ligand stimulation, HER2 over-expression or inhibitor action in a panel of breast cancer cell lines. In matched FFPE sample and cell lysate studies, there is good qualitative agreement between the VeraTag assay and data obtained from traditional Western blotting or co-immunoprecipitation experiments. In addition, we generated a comparable set of data with a cell lysate version of the VeraTag assay (described in Text S1 and Figure S7), which retains the quantitative characteristics of the FFPE assay and provides further cross-validation of the FFPE data. In breast tumors, we confirmed that this panel of analytes can be reproducibly measured at levels above assay background and over a dynamic range consistent with expectation based on cell line models. The positive correlations between HER23D, HER3PI3K and HER3P (Table 1) are expected, as a result of the known signaling in the pathway. Interestingly, the dimerization of HER2 with HER3 appears to be a strong function of HER2 over-expression [36] in this set of tumors, and is a strong indicator of the activation of this pathway, and is likely to be a useful biomarker. The abundance of ligand-independent HER23D is consistent with low HRG expression in high HER2 breast cancer cell lines and tumors [34]. The agreement between HER2T and HER3P measurements is also understandable, since HER2 preferentially phosphorylates HER3 [37] and HER3 receptor is functionally involved in the biology of HER2-positive breast tumors and now represents a new molecular target of therapy [38]. HER23D and HER3PI3K measurements could prove to be useful alternatives to a HER3P biomarker, given their strong covariance.

Frequently, in FFPE samples, protein phosphorylation is used as a marker of pathway activation although the stability of this measurement can be adversely affected by a variety of factors [4]. We measured HER3 phosphorylation in a significant number of well-preserved tumors in this study; however, protocols to preserve target integrity in tumors are not always applied uniformly, especially in a busy pathology laboratory. Therefore, phosphorylation may not be a reliable indicator of pathway signaling in all patient cohorts. In cell lines stimulated with ligand, we observed strong covariance between three key markers of activation, namely, HER3P, HER3PI3K and pAkt. However, in xenograft experiments where fixation of the tumor was intentionally delayed after removal from the animal, we observed a significant degradation of HER3 phosphorylation for the longest time point, while the HER3PI3K complex signal and the HER23D signals were unaffected. We believe that measurement of the HER3PI3K complex could provide a more robust read-out of pathway activation than, for example, HER3 or Akt phosphorylation. It is known that SH2 domains can prevent tyrosine dephosphorylation in vitro [39], which could explain the increased stability of the HER3PI3K complex since the p85 subunit utilizes two SH2 domains to bind to phosphorylated HER3.

We chose three clinically validated HER2 targeting drugs reviewed in [40] to demonstrate that the results of therapeutic intervention in the HER3/PI3K/AKT pathway can be measured with VeraTag FFPE assays. 2C4 is the first of a new class of agents known as HER dimerization inhibitors, which act by binding to HER2, thus preventing dimerization with other HER partners. Trastuzumab (Herceptin) differs from 2C4 in that it is active in high HER2 expressing tumors. Lapatinib is a type I tyrosine kinase inhibitor (TKI) with reactivities towards both EGFR and HER2. Since HER2 is a key determinant of the levels of the HER23 heterodimers and other downstream targets measured in the HER3/PI3K pathway in the breast cancer cell lines we studied, therapeutic inhibition of HER2 should be reflected in the inhibition of this pathway. Response, as measured by pathway activation, was attenuated by the HER-targeted therapies to different degrees, was cell line specific and was dependent on the mode of pathway activation. Basally activated BT474 was inhibited by trastuzumab and lapatinib, but not by 2C4. Although, under the experimental conditions used, we did not observe inhibition of ligand-independent HER23 dimerization in BT474 either by co-immunoprecipitation/Western blotting or by VeraTag assays, there is evidence of pathway inhibition by trastuzumab and especially lapatinib, as pAkt is attenuated. The lack of a measurable effect of trastuzumab on HER23D in BT474 cells was unexpected [13] and is being further investigated.

In ligand activated T474D, 2C4 shows the expected inhibition of HER23D, HER3P and HER3PI3K, consistent with the disruption of HER heterodimers. By contrast, trastuzumab has no effect on these analytes. Lapatinib, however, shows a statistically significant inhibition of HER23D that is unexpected based on known mechanisms of action of TKIs. A possible explanation is that the structural flexibility and stability of inactive-like conformation of HER2 could potentiate lapatinib binding, in a manner seen with lapatinib binding to EGFR [41]. This in turn could lead to the lack of active HER2 available to form HER23D. Surprisingly, for lapatinib and 2C4, upstream pathway inhibition was not accompanied by a decrease in the phosphorylation of Akt. The T47D cell line is known to contain a strongly activating mutation in the kinase domain of PI3-kinase (H1047R), which signals through Akt [42]. This mutation upregulates the phospholipid kinase activity of the protein independent of Ras interaction. Our data indicates that there is residual HER3 phosphorylation at the drug concentrations used under conditions of HRG stimulation, and we speculate that this may be sufficient, when combined with the H1047R mutation in PI3-kinase, to maintain Akt phosphorylation.

In conclusion, we demonstrate the capability of VeraTag assays to quantitatively measure molecular changes in the HER3/PI3K/Akt pathway in both breast cancer cell lines and tumors. Multiple measurements in the pathway, including those that are less dependent on the tissue preservation methodology, should give a more complete picture of pathway status and have greater utility as prognostic or predictive biomarkers. This study provides validation of previous work and extends our knowledge of HER3 complexes in tumors and their responses to selected HER2-targeted therapies. Although the data presented here pertains to the HER family, this assay system is readily adaptable to other protein families for which antibodies or other recognition elements are available due to the universal nature of the assay.

## Supporting Information

Figure S1 **Principle of the VeraTag assay.** Antibodies are added to the FFPE tissue section where they bind to their specific targets. One antibody is labeled with biotin; the other antibody is labeled with a VeraTag. Following the addition of SA-MB, a reactive singlet oxygen species is generated in response to photoactivation and the VeraTag is cleaved from the bound antibody; only VeraTags in proximity to the cleavage agent are released. The cleaved VeraTags from each sample are collected in a single well of a 96-well plate and read on a conventional ABI 3100 CE instrument. The resulting electropherogram is analyzed by the VeraTag Informer software, which identifies the VeraTag peak based on its mobility. Each target measured in the assay is assigned to a unique VeraTag.
(TIF)

Figure S2 **Work-flow of the VeraTag assay.** FFPE slides are dewaxed and hydrated following established IHC protocols (A). Antigen retrieval is done in a pressure cooker (B). Following cooling and addition of blocking buffer, the antibody mix is added to each section (C). The slides are washed, SA-MB added and the VeraTag is cleaved upon illumination (D). The released VeraTag is separated on a CE machine (E) and analyzed using informer software (F).
(TIF)

Figure S3 **Determination of antibody cross-reactivity by immunohistochemistry.** FFPE sections from BT474, HEK 293, HEK 293-HER3 (HEK 293 transfected with HER3) cell lines were immunostained with the indicated antibodies following standard IHC protocols on Ventana Discovery autostainer as described in the Materials and Methods. All images are at 10x magnification. (**A**) Strong-intensity HER3 staining with B9A11. (**B**) Negative control Mo IgG. (**C**) Strong-intensity HER3 staining with SC-285. (**D, E**) Negative control cell lines for HER3. (**F**) Strong HER2 staining intensity with HER2 antibody Ab-8.
(TIF)

Figure S4 **Isotype control and Alkaline Phosphatase experiments.** **A–C**: The indicated cell lines were serum-starved overnight and were either treated with 60 nM HRG (_H) in media for 10 min or left untreated. MDA-MB-231 and NIH-3T3 cells were used without starvation or treatment. The sections were either subjected to the VeraTag FFPE HER23D, HER3PI3K and HER3P assays or the corresponding ITC assays where the biotin-labeled HER3 antibody is replaced with biotin-labeled mouse IgG_1κ_. The normalized RPA values are plotted on the y-axis. Signal from ITC experiment is denoted by the solid bar and assay signal by hatch-marked bars. **D–F**: FFPE sections for each cell line were processed as described in the Materials and Methods and then treated with alkaline phosphatase overnight or left untreated before resuming with the rest of the VeraTag assays. HER23D, HER3P and HER3PI3K assays were performed on the FFPE sections from the panel of 10 breast cancer cell lines with and without HRG stimulation. The normalized RPA values are plotted on the y-axis. Signal from AP treatment is denoted by the solid bar and assay signal by hatch-marked bars.
(TIF)

Figure S5 **Demonstration of precision of the HER23D, HER3P and HER3PI3K assays.** **A–C**: Serial FFPE sections for each cell line were run in the HER23D (A), HER3PI3K(B) and HER3P (C) assays on the same day (eight replicates), as indicated, and the normalized RPA values are plotted on the y-axis.
(TIF)

Figure S6 **Analysis of breast cancer cell line panel.** **A–B**: Total HER2 (top panel) and total HER3 (bottom panel) FFPE assays. The indicated cell lines were serum-starved overnight and were either treated with 60 nM HRG in media for 10 min or left untreated. MDA-MB-231 and NIH-3T3 cells were used without starvation or treatment (refed: RF). FFPE blocks were prepared as described in Materials and Methods. FFPE sections cut from the blocks were used to perform VeraTag assays. Data from the assays were analyzed, and the results are plotted in the graphs. The normalized RPA values are plotted on the y-axis. HRG-stimulated signal is denoted by the solid bars and unstimulated signal by hatch-marked bars. **C–D**: HER2-HER3 heterodimer, phospho-HER3, HER3-PI3K, phospho-Akt analysis of breast cancer cell lines. **C**-Top panel: 50 µg portion of protein extract from the indicated cell lysates were fractionated by SDS-1% PAGE and transferred to a PVDF membrane. The membrane was immunoblotted with HER2, HER3, actin, p-serine 473 Akt, Akt and p85 antibodies. β-Actin is shown for loading control. **D**-Bottom panel: µg portion of protein extract from the indicated cell lysate was immunoprecipitated (IP) with anti-HER3 antibody. Immunoprecipitates were fractionated by SDS-1% PAGE and transferred to a PVDF membrane. The membrane was immunoblotted with HER2, phosphotyrosine and p85 antibodies.
(TIF)

Figure S7 **A–D: Total HER3, HER23D, HER3P and HER3PI3K analysis of breast cancer cell lines by VeraTag lysate assays.** The indicated cell lines were serum-starved overnight and were either treated with 60 nM heregulin in media for 10 min or left untreated. MDA-MB-231 and NIH-3T3 cells were used without starvation or treatment. Cell lysates were prepared in lysis buffer as described in Materials and Methods and protein lysates were quantified. The lysates were subjected to VeraTag lysate assays. Data from the assays were analyzed and the results are plotted in the graphs. The RPA values per mg of protein are plotted on the y-axis. HRG stimulated signal is denoted by the solid bars and unstimulated signal by hatch-marked bars.
(TIF)

Figure S8 **Effects of trastuzumab, 2C4 and lapatinib on molecular markers under HRG-stimulated conditions.** **A**: 300 µg portion of protein extract from the drug treated T47D cell lysates were immunoprecipitated (IP) with biotin-labeled anti-HER3 antibody. Immunoprecipitates were fractionated by SDS-1% PAGE and transferred to a PVDF membrane. The membrane was immunoblotted with anti-HER2, anti-phosphotyrosine and p85 antibodies. Each lane contains equal amounts of immunoprecipitate. Western Blot of the indicated T47D lysates treated with drugs was performed with phospho-Akt and total Akt antibodies after SDS-PAGE and transfer to a PVDF membrane of 50 µg lysate. **B**: VeraTag Akt lysate assay was performed on the indicated T47D lysates treated with drugs (80 µg each). The data is presented as the ratio of phospho-Akt to total Akt.
(TIF)

Figure S9 **Effects of trastuzumab, 2C4 and lapatinib on molecular markers of basally activated breast cancer cells.** **A**: 300 µg portion of protein extract from the drug treated BT474 cell lysates were immunoprecipitated (IP) with biotin-labeled anti-HER3 antibody. Immunoprecipitates were fractionated by SDS-1% PAGE and transferred to a PVDF membrane. The membrane was immunoblotted with anti-HER2, anti-phosphotyrosine and p85 antibodies. Each lane contains equal amounts of immunoprecipitate. Western Blot of the indicated BT474 lysates treated with drugs was performed with phospho-Akt and total Akt antibodies after SDS-PAGE and transfer to a PVDF membrane of 50 µg lysate. **B**: VeraTag Akt lysate assay was performed on the indicated BT474 lysates treated with drugs ( µg each). The data is presented as the ratio of phospho-Akt to total Akt.
(TIF)

Figure S10 **A–B: HER2 and HER3 levels are not affected by dephosphorylation upon delayed fixation in xenograft.** Tumors sections from the five groups of mice were subjected to the VeraTag FFPE HER2T and HER3T assays. The normalized RPA values are plotted on the y-axis.
(TIF)

Text S1 **Details of the VeraTag assay technology, protocols and assay characterization.**
(DOC)

Table S1 **Flow cytometric analysis of HER2 and HER3 receptors are reported as number of receptors per cell for all the cell lines used in the study.**
(DOC)
