# Supplementary figures and images for: Profiling the HER3/PI3K Pathway in Breast Tumors Using Proximity-Directed Assays Identifies Correlations between Protein Complexes and Phosphoproteins
Source: PLoS One. 2011 Jan 28;6(1):e16443. doi: 10.1371/journal.pone.0016443 (PMC3030586; doi:10.1371/journal.pone.0016443)

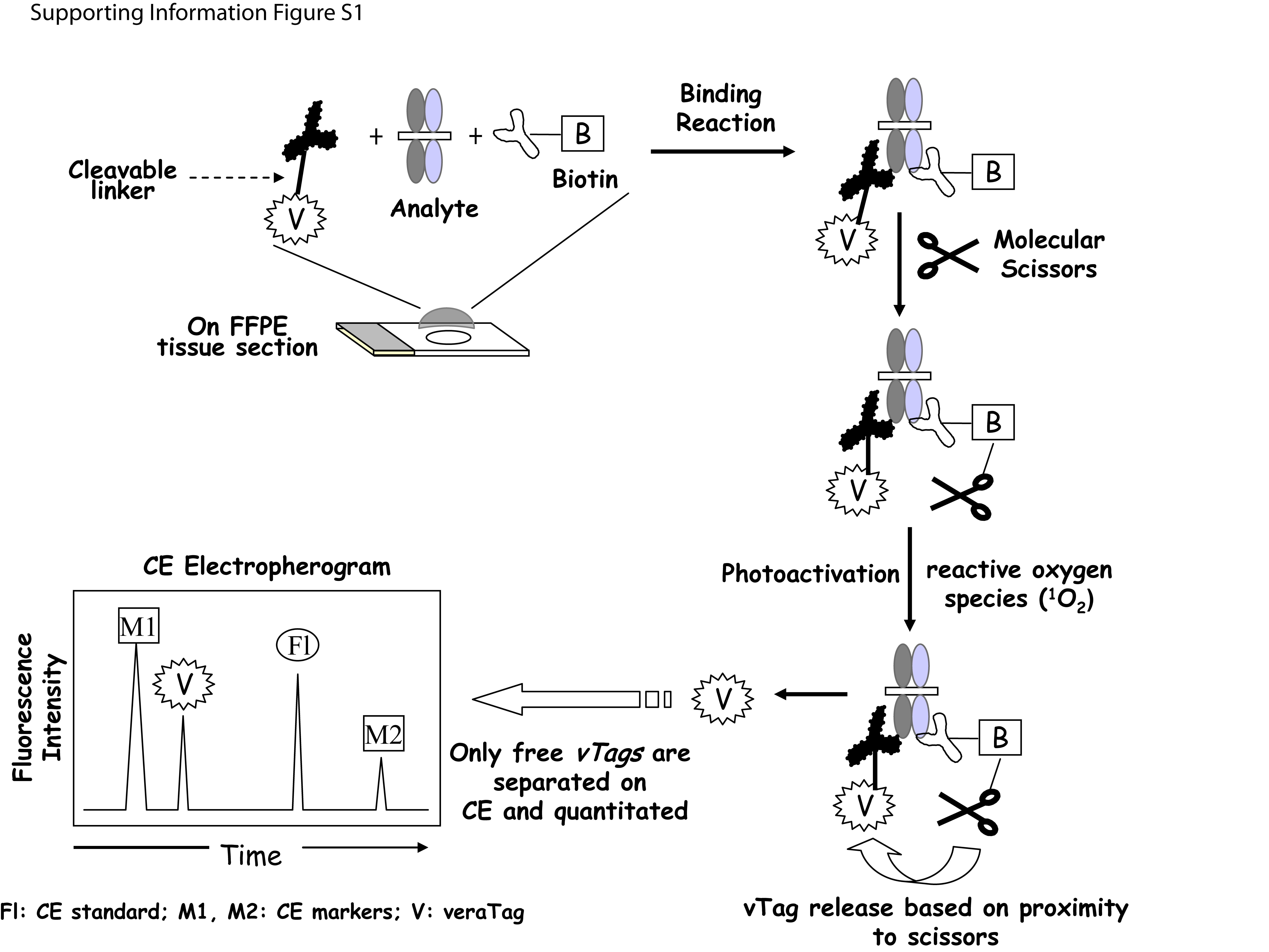

Supplement: Figure S1 — Principle of the VeraTag assay. Antibodies are added to the FFPE tissue section where they bind to their specific targets. One antibody is labeled with biotin; the other antibody is labeled with a VeraTag. Following the addition of SA-MB, a reactive singlet oxygen species is generated in response to photoactivation and the VeraTag is cleaved from the bound antibody; only VeraTags in proximity to the cleavage agent are released. The cleaved VeraTags from each sample are collected in a single well of a 96-well plate and read on a conventional ABI 3100 CE instrument. The resulting electropherogram is analyzed by the VeraTag Informer software, which identifies the VeraTag peak based on its mobility. Each target measured in the assay is assigned to a unique VeraTag. (TIF) [file pone.0016443.s001.tif]

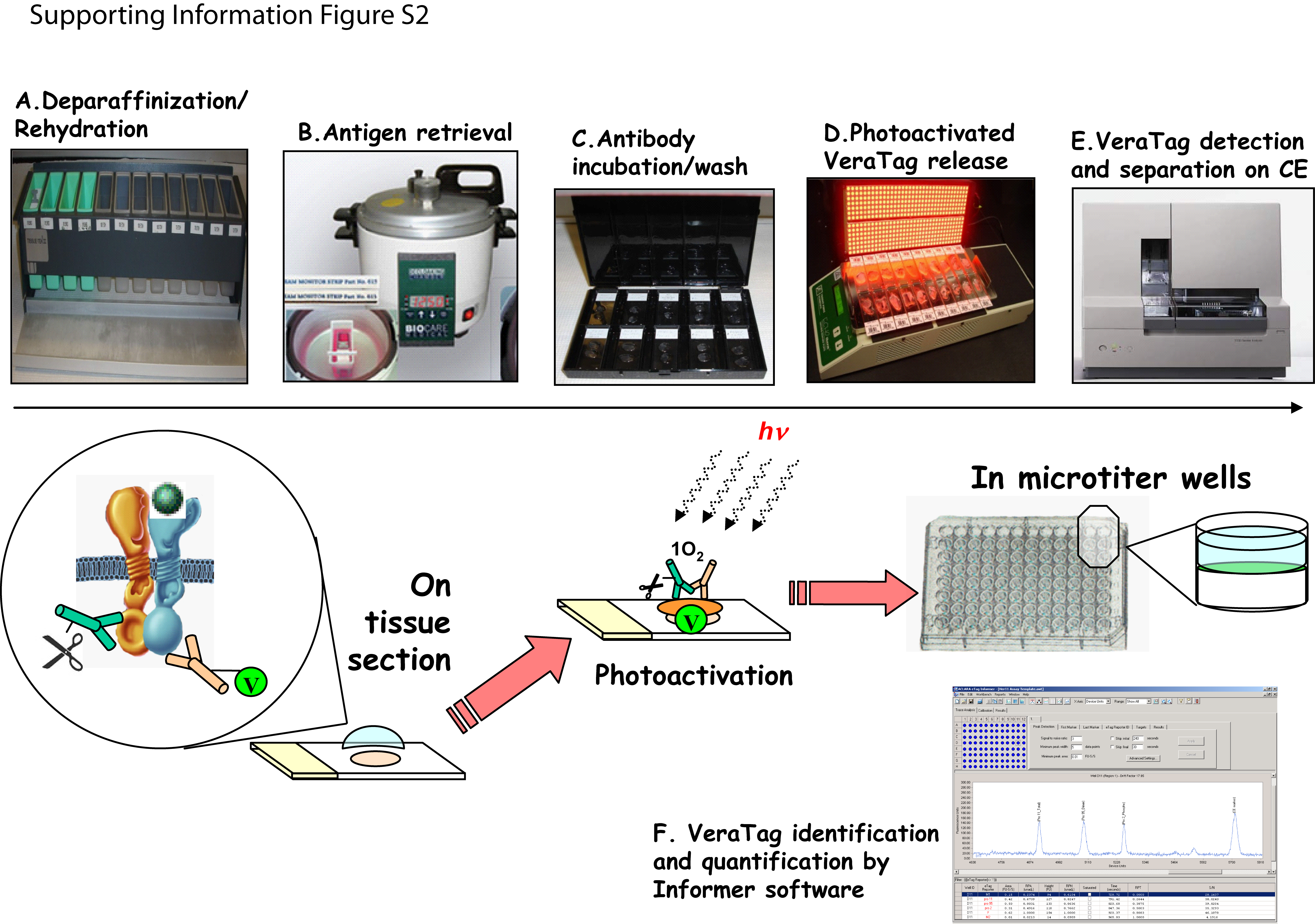

Supplement: Figure S2 — Work-flow of the VeraTag assay. FFPE slides are dewaxed and hydrated following established IHC protocols (A). Antigen retrieval is done in a pressure cooker (B). Following cooling and addition of blocking buffer, the antibody mix is added to each section (C). The slides are washed, SA-MB added and the VeraTag is cleaved upon illumination (D). The released VeraTag is separated on a CE machine (E) and analyzed using informer software (F). (TIF) [file pone.0016443.s002.tif]

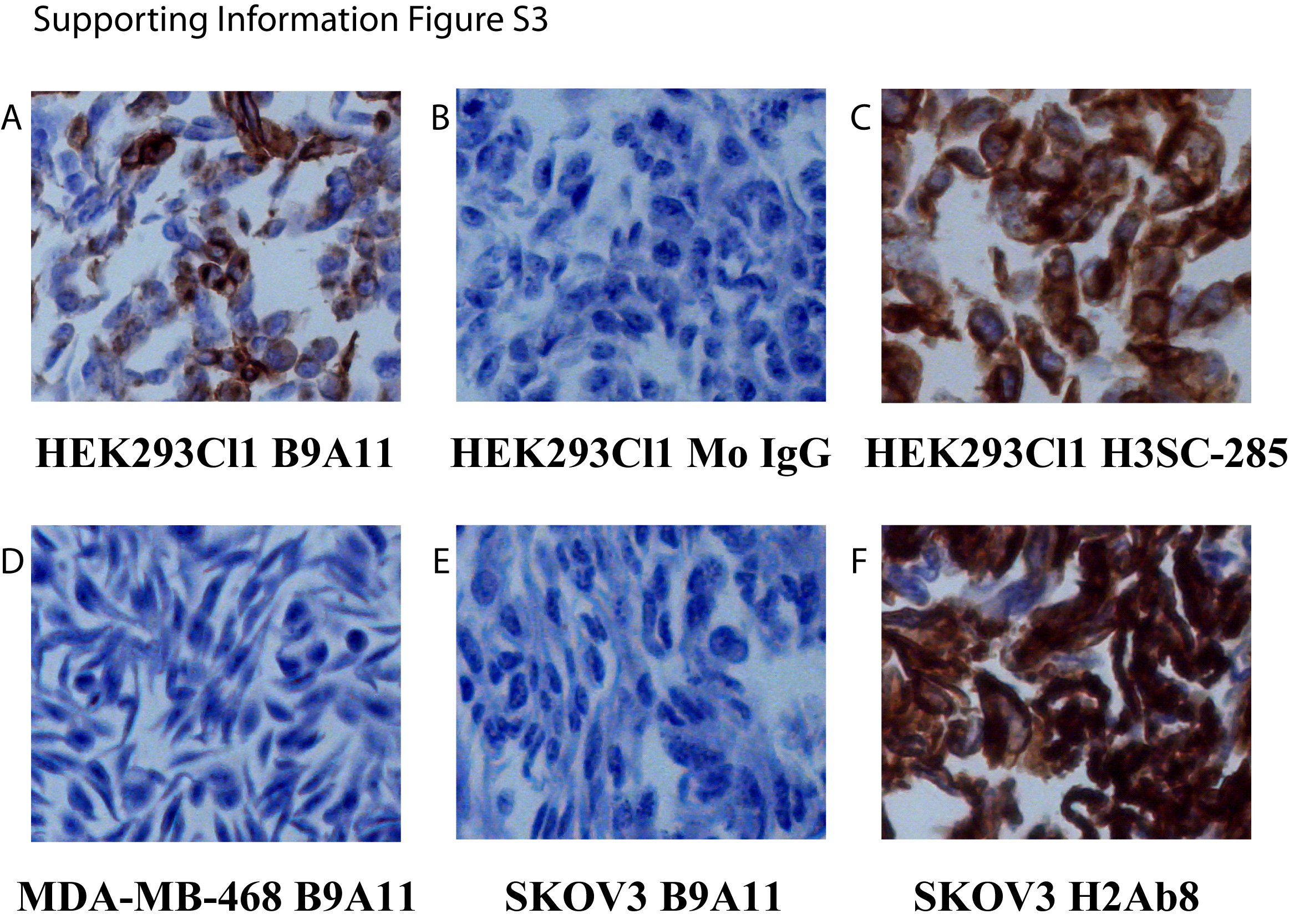

Supplement: Figure S3 — Determination of antibody cross-reactivity by immunohistochemistry. FFPE sections from BT474, HEK 293, HEK 293-HER3 (HEK 293 transfected with HER3) cell lines were immunostained with the indicated antibodies following standard IHC protocols on Ventana Discovery autostainer as described in the Materials and Methods. All images are at 10x magnification. (A) Strong-intensity HER3 staining with B9A11. (B) Negative control Mo IgG. (C) Strong-intensity HER3 staining with SC-285. (D, E) Negative control cell lines for HER3. (F) Strong HER2 staining intensity with HER2 antibody Ab-8. (TIF) [file pone.0016443.s003.tif]

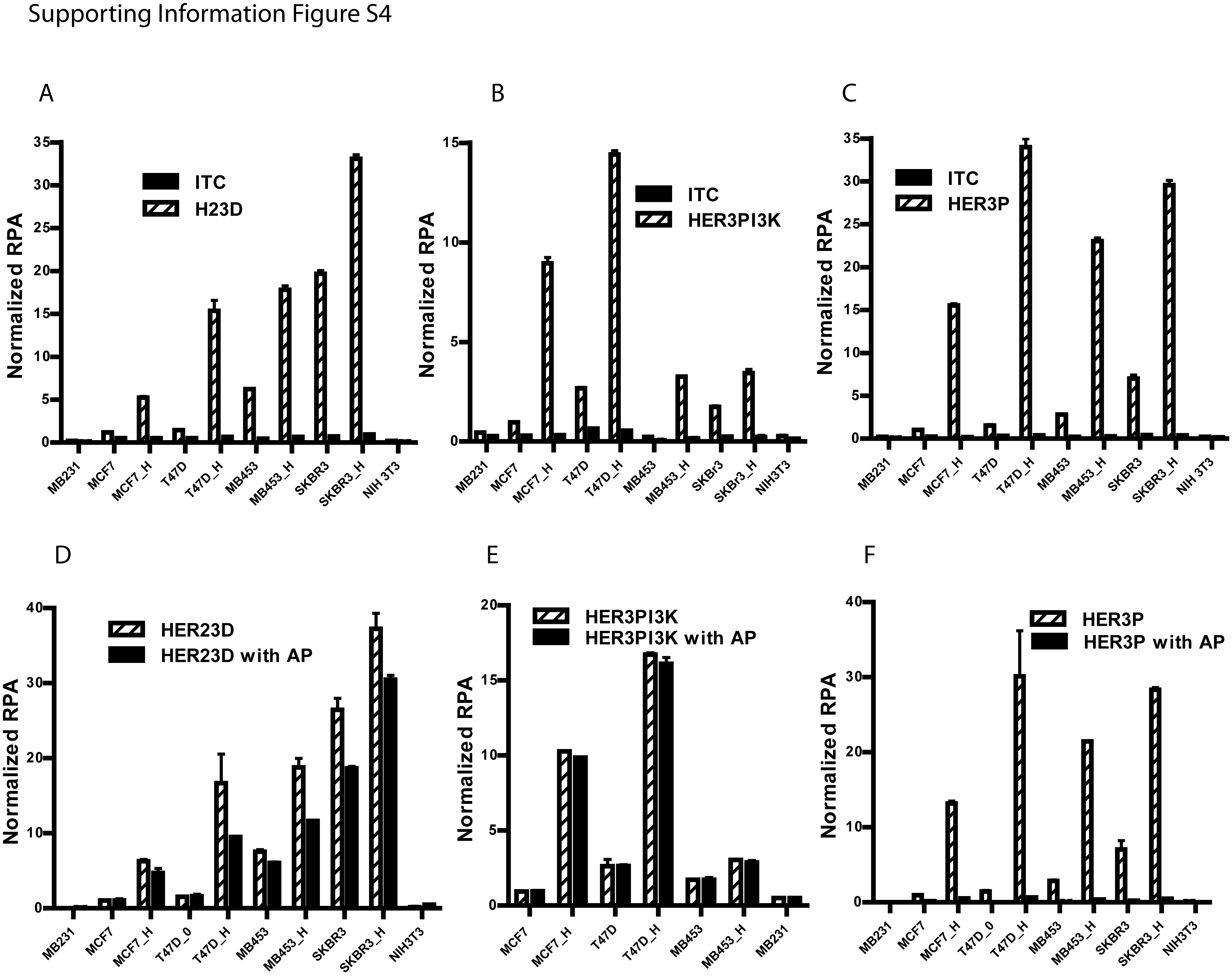

Supplement: Figure S4 — Isotype control and Alkaline Phosphatase experiments. A–C: The indicated cell lines were serum-starved overnight and were either treated with 60 nM HRG (_H) in media for 10 min or left untreated. MDA-MB-231 and NIH-3T3 cells were used without starvation or treatment. The sections were either subjected to the VeraTag FFPE HER23D, HER3PI3K and HER3P assays or the corresponding ITC assays where the biotin-labeled HER3 antibody is replaced with biotin-labeled mouse IgG1κ. The normalized RPA values are plotted on the y-axis. Signal from ITC experiment is denoted by the solid bar and assay signal by hatch-marked bars. D–F: FFPE sections for each cell line were processed as described in the Materials and Methods and then treated with alkaline phosphatase overnight or left untreated before resuming with the rest of the VeraTag assays. HER23D, HER3P and HER3PI3K assays were performed on the FFPE sections from the panel of 10 breast cancer cell lines with and without HRG stimulation. The normalized RPA values are plotted on the y-axis. Signal from AP treatment is denoted by the solid bar and assay signal by hatch-marked bars. (TIF) [file pone.0016443.s004.tif]

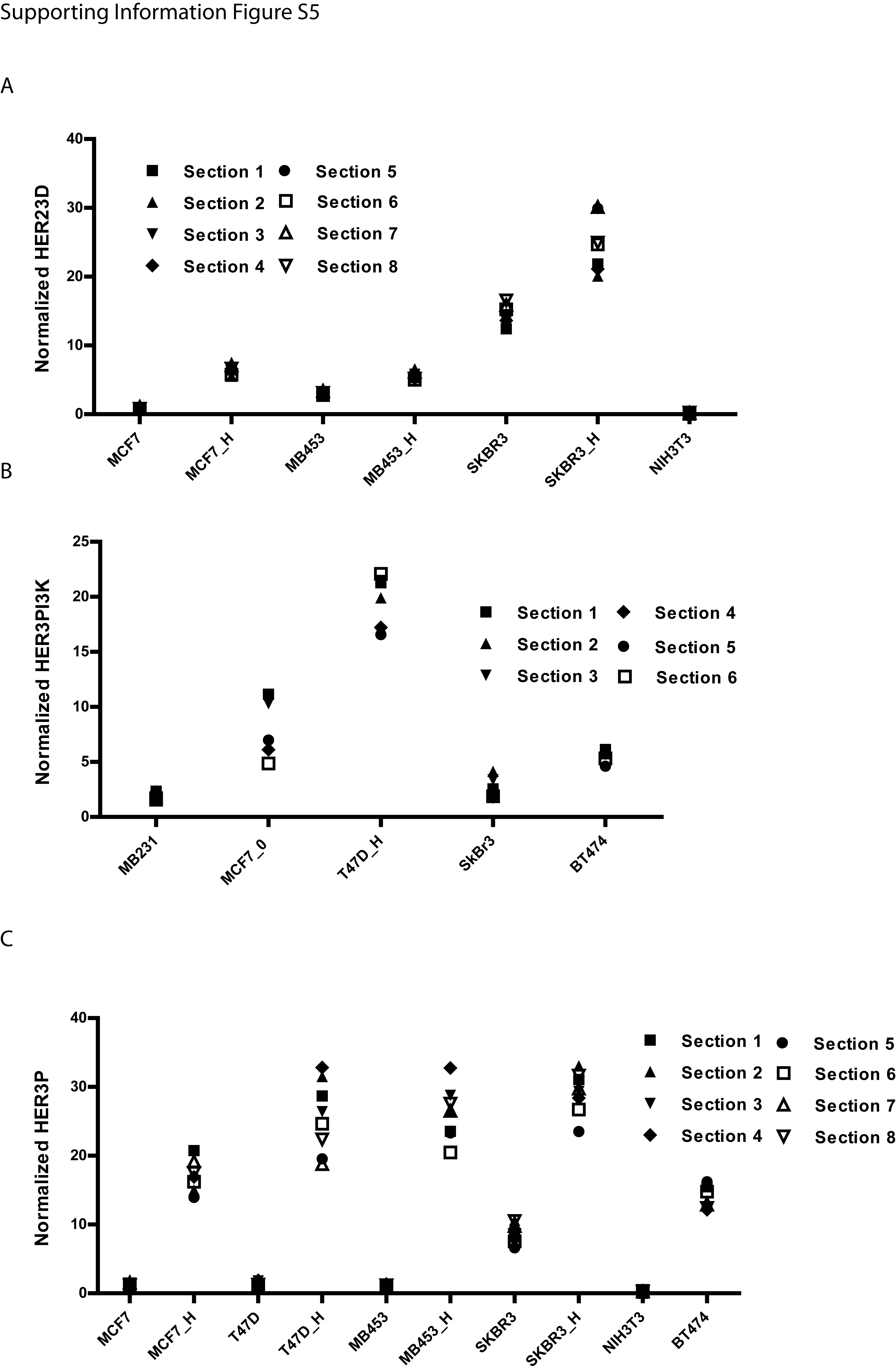

Supplement: Figure S5 — Demonstration of precision of the HER23D, HER3P and HER3PI3K assays. A–C: Serial FFPE sections for each cell line were run in the HER23D (A), HER3PI3K(B) and HER3P (C) assays on the same day (eight replicates), as indicated, and the normalized RPA values are plotted on the y-axis. (TIF) [file pone.0016443.s005.tif]

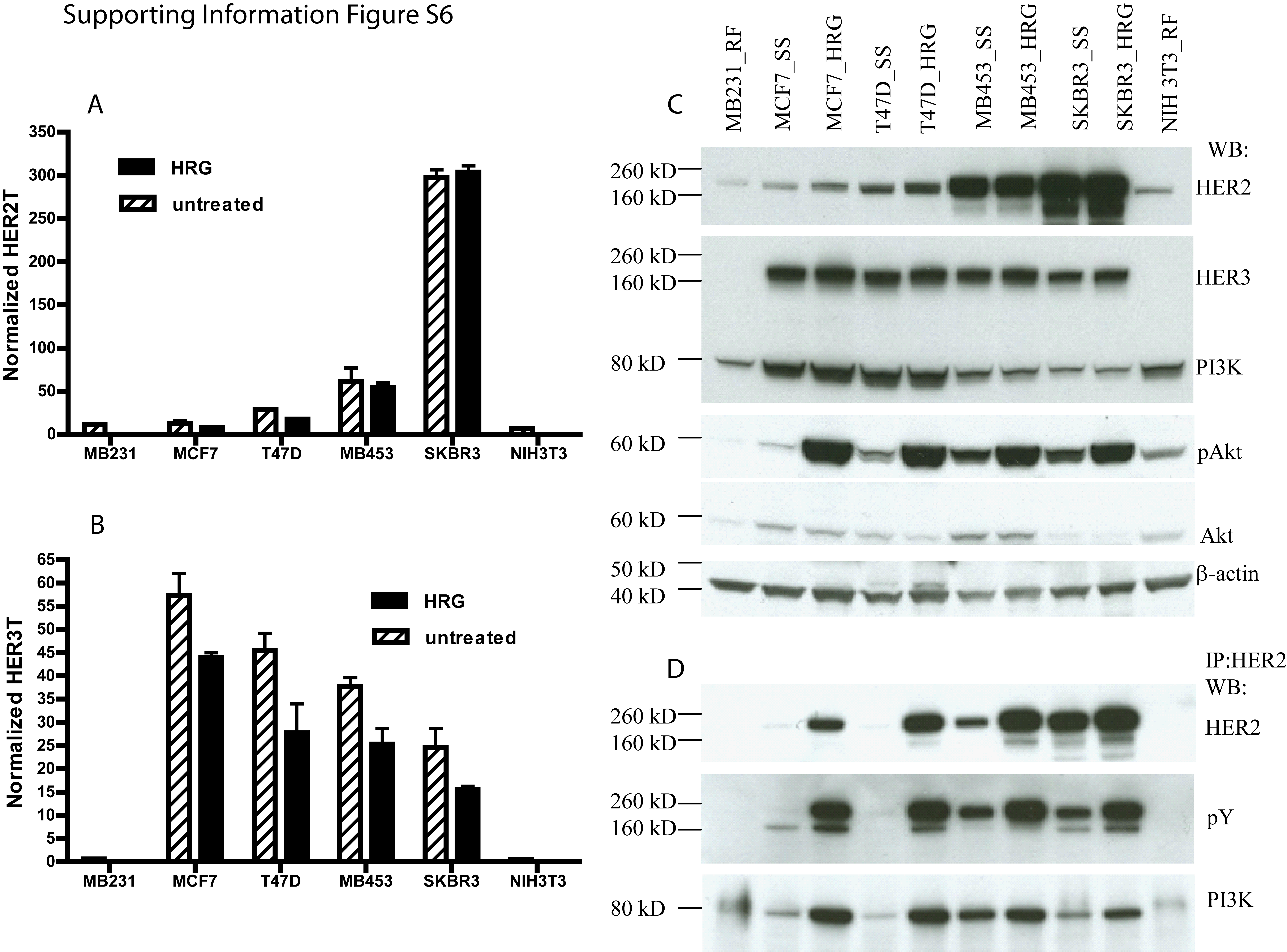

Supplement: Figure S6 — Analysis of breast cancer cell line panel. A–B: Total HER2 (top panel) and total HER3 (bottom panel) FFPE assays. The indicated cell lines were serum-starved overnight and were either treated with 60 nM HRG in media for 10 min or left untreated. MDA-MB-231 and NIH-3T3 cells were used without starvation or treatment (refed: RF). FFPE blocks were prepared as described in Materials and Methods. FFPE sections cut from the blocks were used to perform VeraTag assays. Data from the assays were analyzed, and the results are plotted in the graphs. The normalized RPA values are plotted on the y-axis. HRG-stimulated signal is denoted by the solid bars and unstimulated signal by hatch-marked bars. C–D: HER2-HER3 heterodimer, phospho-HER3, HER3-PI3K, phospho-Akt analysis of breast cancer cell lines. C-Top panel: 50 µg portion of protein extract from the indicated cell lysates were fractionated by SDS-1% PAGE and transferred to a PVDF membrane. The membrane was immunoblotted with HER2, HER3, actin, p-serine 473 Akt, Akt and p85 antibodies. β-Actin is shown for loading control. D-Bottom panel: µg portion of protein extract from the indicated cell lysate was immunoprecipitated (IP) with anti-HER3 antibody. Immunoprecipitates were fractionated by SDS-1% PAGE and transferred to a PVDF membrane. The membrane was immunoblotted with HER2, phosphotyrosine and p85 antibodies. (TIF) [file pone.0016443.s006.tif]

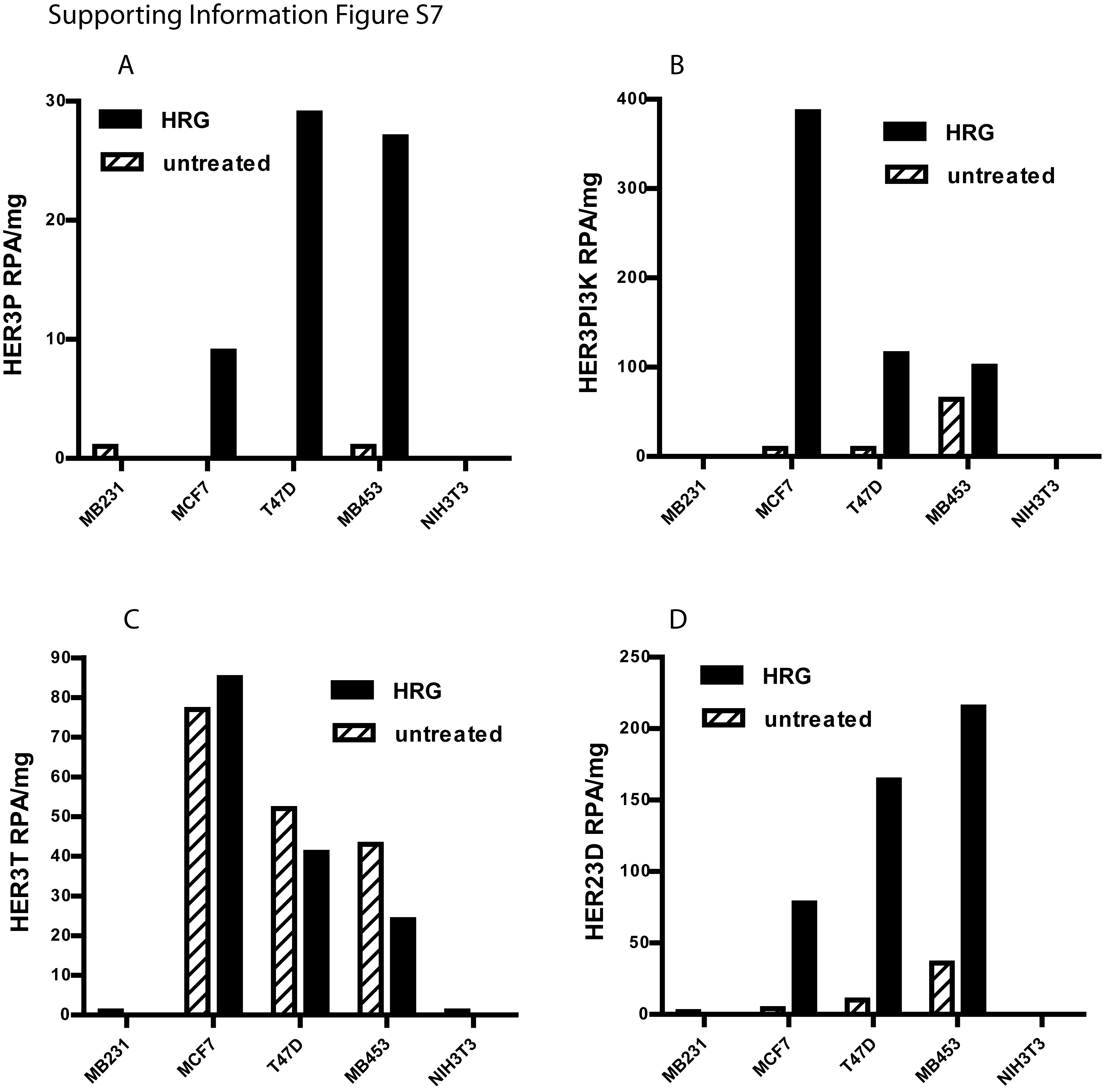

Supplement: Figure S7 — A–D: Total HER3, HER23D, HER3P and HER3PI3K analysis of breast cancer cell lines by VeraTag lysate assays. The indicated cell lines were serum-starved overnight and were either treated with 60 nM heregulin in media for 10 min or left untreated. MDA-MB-231 and NIH-3T3 cells were used without starvation or treatment. Cell lysates were prepared in lysis buffer as described in Materials and Methods and protein lysates were quantified. The lysates were subjected to VeraTag lysate assays. Data from the assays were analyzed and the results are plotted in the graphs. The RPA values per mg of protein are plotted on the y-axis. HRG stimulated signal is denoted by the solid bars and unstimulated signal by hatch-marked bars. (TIF) [file pone.0016443.s007.tif]

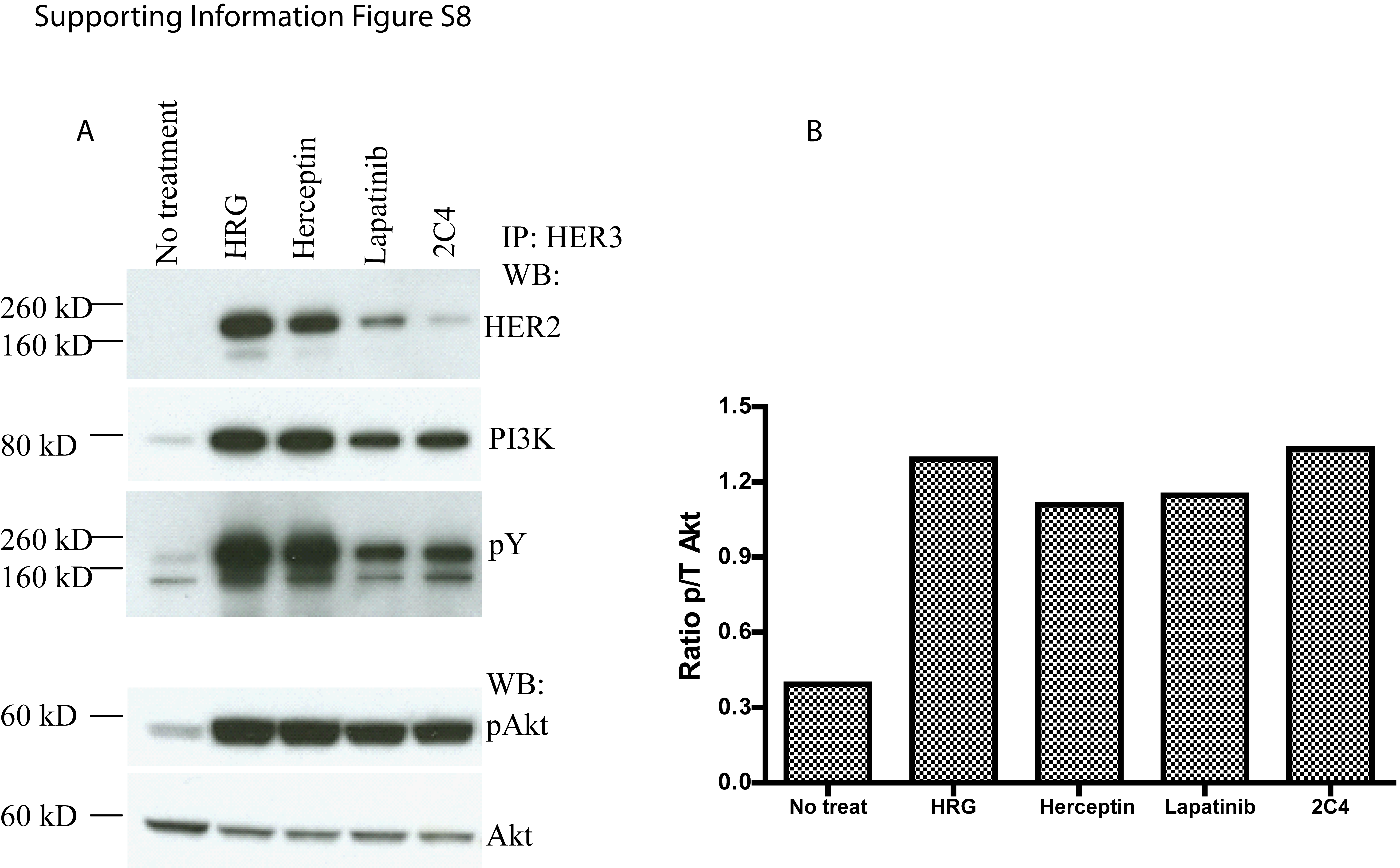

Supplement: Figure S8 — Effects of trastuzumab, 2C4 and lapatinib on molecular markers under HRG-stimulated conditions. A: 300 µg portion of protein extract from the drug treated T47D cell lysates were immunoprecipitated (IP) with biotin-labeled anti-HER3 antibody. Immunoprecipitates were fractionated by SDS-1% PAGE and transferred to a PVDF membrane. The membrane was immunoblotted with anti-HER2, anti-phosphotyrosine and p85 antibodies. Each lane contains equal amounts of immunoprecipitate. Western Blot of the indicated T47D lysates treated with drugs was performed with phospho-Akt and total Akt antibodies after SDS-PAGE and transfer to a PVDF membrane of 50 µg lysate. B: VeraTag Akt lysate assay was performed on the indicated T47D lysates treated with drugs (80 µg each). The data is presented as the ratio of phospho-Akt to total Akt. (TIF) [file pone.0016443.s008.tif]

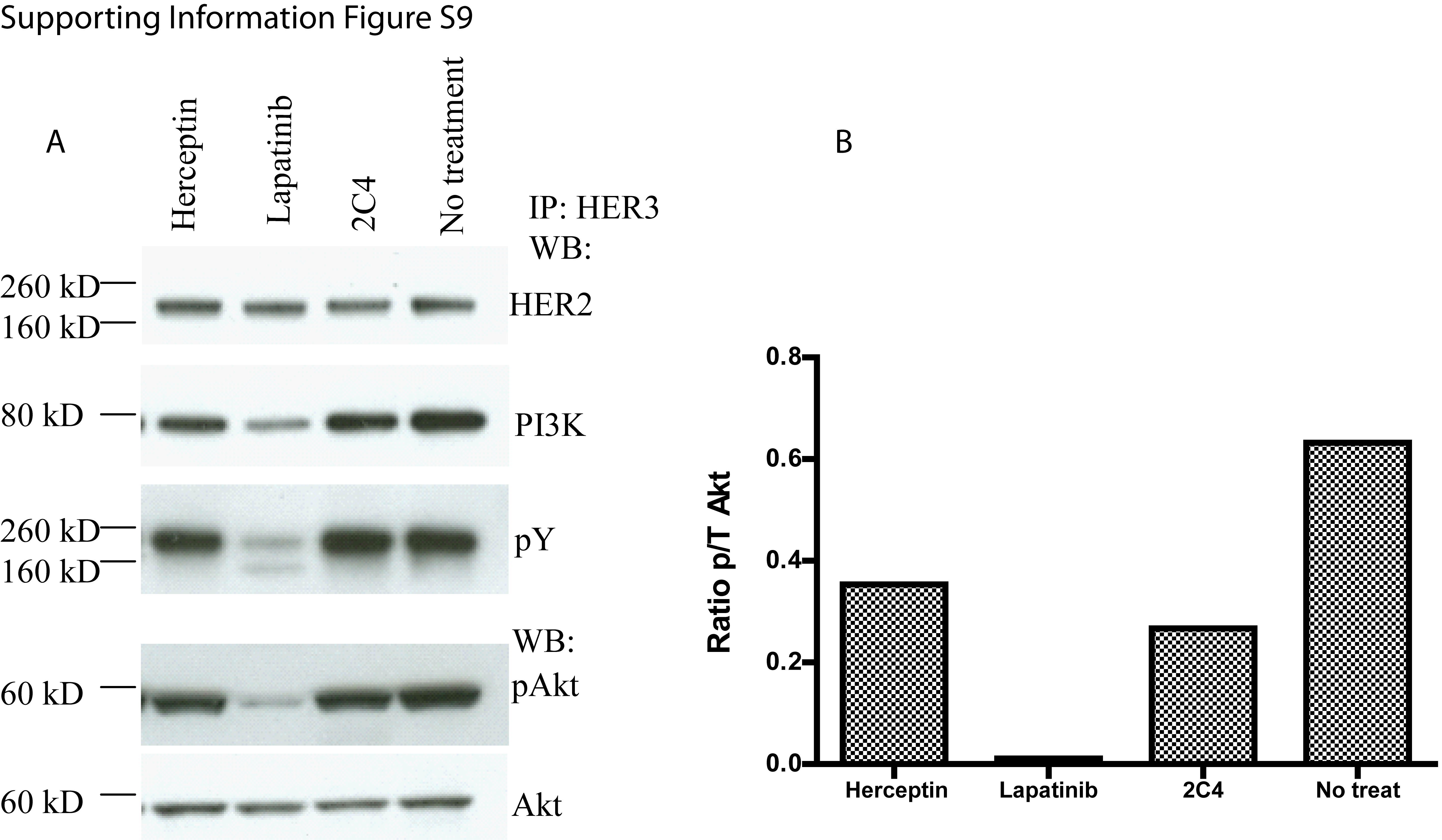

Supplement: Figure S9 — Effects of trastuzumab, 2C4 and lapatinib on molecular markers of basally activated breast cancer cells. A: 300 µg portion of protein extract from the drug treated BT474 cell lysates were immunoprecipitated (IP) with biotin-labeled anti-HER3 antibody. Immunoprecipitates were fractionated by SDS-1% PAGE and transferred to a PVDF membrane. The membrane was immunoblotted with anti-HER2, anti-phosphotyrosine and p85 antibodies. Each lane contains equal amounts of immunoprecipitate. Western Blot of the indicated BT474 lysates treated with drugs was performed with phospho-Akt and total Akt antibodies after SDS-PAGE and transfer to a PVDF membrane of 50 µg lysate. B: VeraTag Akt lysate assay was performed on the indicated BT474 lysates treated with drugs ( µg each). The data is presented as the ratio of phospho-Akt to total Akt. (TIF) [file pone.0016443.s009.tif]

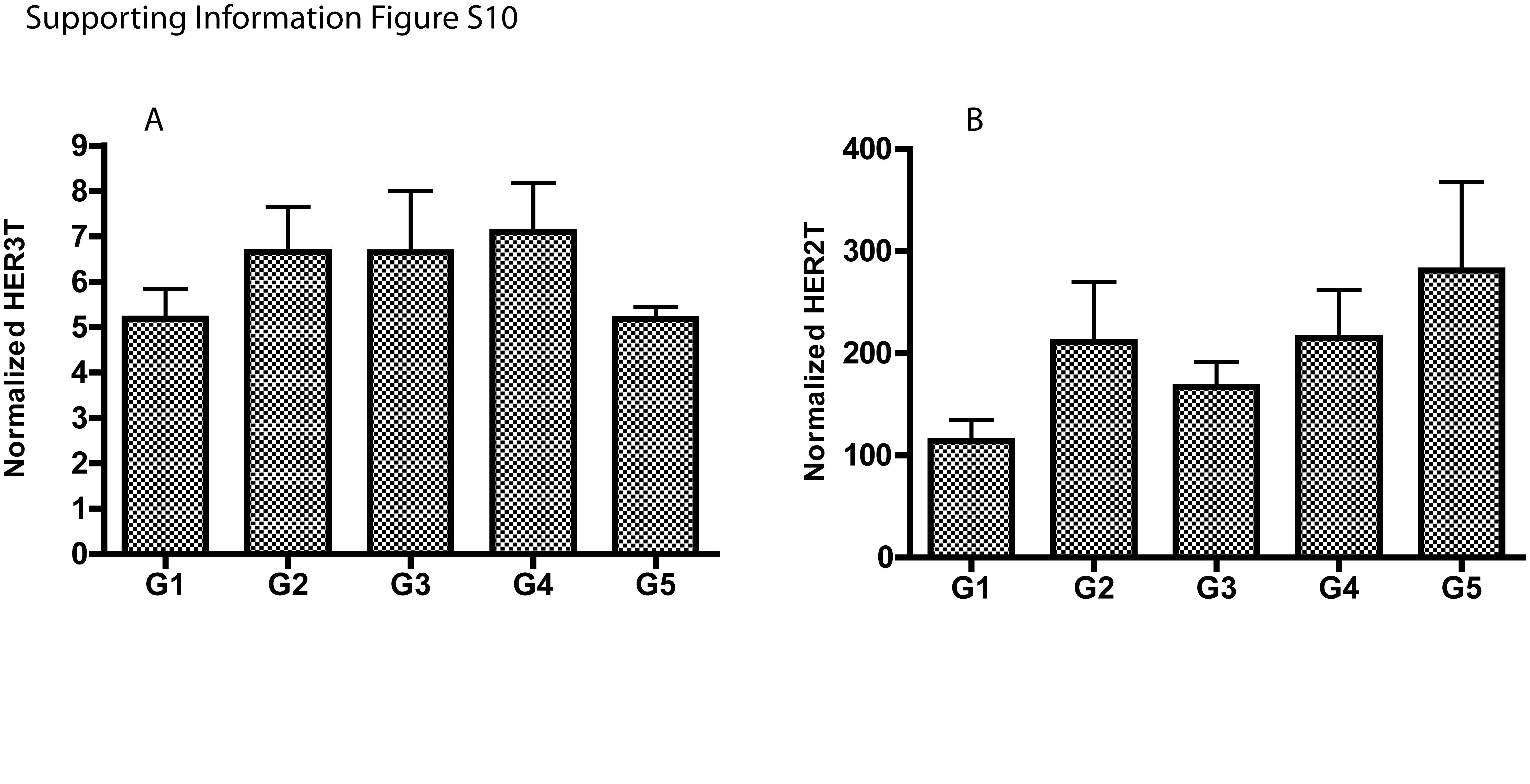

Supplement: Figure S10 — A–B: HER2 and HER3 levels are not affected by dephosphorylation upon delayed fixation in xenograft. Tumors sections from the five groups of mice were subjected to the VeraTag FFPE HER2T and HER3T assays. The normalized RPA values are plotted on the y-axis. (TIF) [file pone.0016443.s010.tif]
