# Supplementary material for: Profiling the HER3/PI3K Pathway in Breast Tumors Using Proximity-Directed Assays Identifies Correlations between Protein Complexes and Phosphoproteins
Source: PLoS One. 2011 Jan 28;6(1):e16443. doi: 10.1371/journal.pone.0016443 (PMC3030586; doi:10.1371/journal.pone.0016443)
Supplement: Text S1 — Details of the VeraTag assay technology, protocols and assay characterization. (DOC) [file pone.0016443.s011.doc]

**Profiling the HER3/PI3K pathway in breast tumors using Proximity-Directed Assays identifies correlations between protein complexes and phosphoproteins**

**Ali Mukherjee, Youssouf Badal et al.**

Monogram Biosciences

**Text S1: Supporting information Data**

***Background of the Technology:***

The principle and workflow of the VeraTag assay is described in Figure 1 and in the supporting information Figures S1-S2. Formalin-fixed, paraffin-embedded cell line or tissue sections on positively charged glass slides are deparaffinized in xylene and hydrated. In the antibody mix, VeraTag-labeled antibody specific to one epitope of the target was used in conjunction with a biotin-labeled antibody specific to a second distinct epitope of the target. Following antigen retrieval and antibody incubation, the photosensitizer (‘molecular scissors’) streptavidin methylene blue (SA-MB) is added to the tissue section, which binds to the biotin-labeled antibody. Photoactivation releases the reactive singlet oxygen, which cleaves the VeraTag (covalently linked to the antibody via a photocleavable linker) in close proximity to the biotin-labeled antibody. The released VeraTag is collected and separated using capillary electrophoresis (CE) and analyzed and quantitated with the VeraTag Informer software.

***Materials and Method:***

*Antibody conjugation:* Each antibody used in the VeraTag™ assays was conjugated either to a VeraTag reporter or to biotin; which binds to the ‘molecular scissors’ in the assay. The VeraTag molecules were synthesized as NHS ester derivatives following standard synthetic organic chemistry protocols (U.S. Patent 7,105,308). Sulfo-NHS-LC-LC biotin was purchased from Pierce. The antibodies were first purified using either a desalting column (PD-10) or a protein-G column followed by buffer exchange. The antibody conjugation was performed according to standard conjugation chemistry employing NHS esters of VeraTag or biotin. The conjugate was purified on a size exclusion column followed by the addition of preservatives and stabilizers. In a typical reaction, 1 mg of antibody was added to 100 nmoles of VeraTag NHS ester in 1 mL PBS. After 1 h on ice, the reaction was quenched with 100 µL of 100 mM glycine. The reaction mixture was then purified on a G-50 column using HPLC (Agilent) using PBS as eluant. The concentration and the hapten number of the conjugate were calculated from the absorbance at 280 nm and 495 nm. The conjugate was stored at 4 °C after the addition of bovine serum albumin (BSA) and sodium azide.

*Molecular scissors:* NHS derivative of methylene blue was synthesized using standard synthetic organic methodologies and reacted with commercially available streptavidin (Roche) to obtain streptavidin-methylene blue. The resulting product was purified by size exclusion chromatography using a sephadex G-50 column.

*Cell culture and stimulation:* All cancer cell lines were purchased from ATCC and cultured for two weeks at 37 °C and 5% CO_2_ in DMEM+F12 medium supplemented with 10% (v/v) heat inactivated fetal bovine serum and 1% (v/v) penicillin/streptomycin. The cultures were maintained in 6-well culture plates for experiments to determine optimal growth factor concentration and time of stimulation. Prior to use, the cells at 75-85% confluence were starved of serum overnight by replacing the above medium with serum free DMEM+F12 medium. The following day, the serum-free medium was replaced with fresh serum-free medium. Each well was used for a single dose of ligand stimulation. Heregulin (HRG) doses (0-100 nM) were prepared fresh and added to the cells for 0-120 min. After the treatment, the medium was aspirated out while keeping the culture plate on ice. The cells were washed with 1x PBS containing 1mM sodium vanadate. Subsequently, lysis buffer was added to prepare lysates or neutral buffered formalin (NBF) was added to make formalin-fixed, paraffin-embedded (FFPE) blocks. When preparing FFPE blocks, the cells were treated with a single dose of HRG (60 nM) at a single time point (10 min) and grown in 500 cm^2^ plates.

*Fluorescence Activated Cell Sorting (FACS) analysis of cell surface ErbB receptor expression:* Cells were harvested by trypsinization and counted. Approximately 0.5 million cells were labeled with biotinylated monoclonal mouse anti-human ErbB1/ (EGFR Ab-11; Lab Vision), ErbB2 (HER2 Ab-5; Lab Vision) or ErbB3 (HER3 IB4C3; Santa Cruz.) antibodies, at concentrations of 4 µg/mL in total volume of 100 µL. All assays included antibody isotype controls. Cells and antibodies were incubated on ice for 45 min, and cells were washed twice with PBS, followed by labeling with R-phycoerythrin (PE) -avidin (Molecular Probes) at a concentration of 2 µg/mL for 30 min. The labeled cells were washed with PBS twice before fixing with 1% paraformaldehyde in 1x PBS. FACS analysis was carried on FACS Calibur cytometer (BD Biosciences). R-PE fluorescence intensity of labeled cells was determined on FL2 (585/42 nm band pass filter) detector. The quantitation of the fluorescence intensity of the sample in terms of number of molecules of ErbB receptors was calculated based on a calibrated standard curve using Quantum PE MESF Kit (Bangs Laboratories, Inc).

*FFPE block preparation*: After fixation with NBF, cells were harvested by scraping and collected in labeled bottles. They were centrifuged at 14,000 g for 5 min. The resulting pellets were packed into a plastic O-ring and a cassette prior to processing on a Tissue Tek processor. Frozen breast tumor tissues (Asterand) and xenograft samples (Charles River) were fixed in NBF in labeled cassettes overnight followed by processing as described for cell lines. The cell pellets and tumors were embedded in paraffin, and 5-micron sections were cut and placed on positively charged glass slides (VWR).

*Slide pre-treatment:* FFPE sections on glass slides were de-paraffinized using xylene following an established protocol. Antigen retrieval was performed either by boiling the slides in citrate buffer (pH 6.4) in a microwave for 10 min or in DAKO buffer (pH 9.0) in a pressure cooker (Biocare Medical) following their protocol. This was followed by pre-blocking of the tissue with blocking buffer for 1 h at room temperature. The blocking buffer contains 10% normal goat serum and 1.5% BSA in PBS with protease and phosphatase inhibitors.

*Receptor dimerization and phosphorylation FFPE assays:* VeraTag-labeled and biotinylated antibodies were added to the FFPE sections in the blocking buffer (100 µL per section) for 14 h at 4 ^o^C. Receptor phosphorylation was determined by analyzing the VeraTags released in a standard VeraTag proximity assay using VeraTag-labeled anti-phospho tyrosine antibody and biotinylated anti-receptor specific antibody. For heterodimer detection, VeraTag-labeled antibody specific to one member of the dimer was used, in conjunction with a biotin-labeled antibody specific to the second member. The labeled antibodies were used at a final concentration of 1 µg/mL in the blocking buffer. After incubation with appropriate antibodies, the slides were rinsed, and the sections were incubated with streptavidin-labeled molecular scissors (2.5 µg per section) for 1 h at room temperature. The slides were rinsed with 1xPBS and 100 µL illumination buffer (containing 2 pM fluorescein and two CE migration markers) was added to the sections, followed by illumination using Monogram Bioscience’s illumination device (~680 nm LED light, for 1 h, with samples maintained at 4 ^o^C). The illumination buffer containing the released VeraTag molecules was analyzed by capillary electrophoresis (using ABI 3100 genetic analyzer; Applied Biosystems). Fluorescein was used as a normalization standard for all CE runs. For FFPE assays with alkaline phosphatase (AP), following slide pre-treatment, 0.1 U/50 µL of calf-intestinal AP were added to the control sections and incubated overnight. Following 5x washes with PBS and antibody addition, the slides were processed as before.

*Immunohistochemistry and H&E staining:* All IHC was performed on the Ventana Discovery XT following the manufacturer’s protocol with some modifications. HER2 IHC was done using the CB11 antibody (Ventana Medical) for 1 h, HER3 IHC was done using SC-285 or B9A11 (2 µg/mL). All slides underwent staining with haematoxylin and eosin (H&E) after the completion of VeraTag assays followed by cover slipping in permount.

*Estimation of tumor area:* The cover-slipped H&E slides were scanned on a commercial flatbed scanner and the section areas were measured using Image J software (<http://rsb.info.nih.gov/ij/>). In the case of tumor samples, a pathologist circled areas of invasive carcinoma on the slide and the tumor area was subsequently measured using an image analysis software. Samples with significant necrosis, insignificant tumor content or a large non-invasive tumor component, e.g., ductal carcinoma in situ (DCIS) were excluded from analysis. Non-tumor stromal cells do not produce VeraTag signal and are therefore considered non-interfering tissue for the purposes of our analysis.

*Preparation of cell lysates:* Lysates were generated in detergent-containing lysis buffer for protein analysis. Cold lysis buffer was added directly to the cells in the cell culture dish on ice to lyse cells in-situ and the plate was swirled to distribute the lysis buffer evenly. The cells were scraped and transferred to a labeled 1.5 mL microfuge tube. The tube was briefly vortexed at maximal setting and incubated on ice for 15-20 min. Nuclei and detergent-insoluble materials were removed by centrifugation at 14,000 rpm at 4 °C for 10 min. The supernatant was then transferred in 50 µL aliquots to labeled fresh tubes or strip-wells and frozen at –80 °C. A 10 µL aliquot was set aside for BCA assays.

Cells grown in P10 dishes (10 cm diameter) were lysed in 250 µL lysis buffer, and 60 µL of lysis buffer was added to each well of a 6-well plate to maximize the protein concentration in lysates. Protein concentration was determined using BCA reagents (Pierce). Cell culture at 80% confluence yields ~4-6 mg/mL total proteins depending on cell lines. Detergent-containing lysis buffer A contains 1% Triton X-100, 50 mM Tris-HCl (pH 7.5), 35 mM NaCl, 50 mM NaF, 50 mM sodium beta-glycerophosphate, 1 mM Na_3_VO_4_, 5 mM EDTA and 1 tablet (per 10 ml) complete protease inhibitor (Roche) in de-ionized water.

*SDS-PAGE and Western blot analysis:* For HER3 immunoprecipitation, C-terminal anti- HER3 antibody SC-285 (Santa Cruz) was incubated with protein G beads (Pierce) in 100 mM HEPES (pH 8.2), 150 mM NaCl buffer at 4 °C for 2 h. The beads were washed and 300 µg of lysate protein was immunoprecipitated overnight at 4 °C with 20 µL antibody-protein G beads. After immunoprecipitation, the beads were washed twice with lysis buffer. SDS-sample buffer containing an additional 50 mM dithiothreitol was added to each precipitate, and the samples were boiled for 4 min. The samples were run on a 4-12% bis-tris gel and transferred to a PVDF membrane. Coimmunoprecipitating HER2, p85 and phosphoproteins were analyzed by Western blot with Ab-8, 4G10 or 05-212 antibodies respectively. When 2C4- or 4D5-treated samples were analyzed, biotin labeled HER3 SC-285 was incubated with the lysates at 4 °C overnight. Streptavidin-coated beads (Pierce) were used for immunoprecipitation for 2 h at 4 °C. Following immunoprecipitation, the samples were processed as before. Western blotting for Akt and phospho-Akt proteins were performed after SDS-PAGE and transfer to PVDF membrane using SC-5298 (Santa Cruz) and 4051 (CST) antibodies respectively, using manufacturer recommended dilutions.

*Receptor dimerization and phosphorylation lysate assays:* For multiplexed receptor phosphorylation, protein complex and heterodimer detection, VeraTag- and biotin- labeled antibodies specific to the protein dimer/complex and phosphotyrosine were used in a multiplex assay. The molecular scissors-beads (streptavidin-coated phthalocyanin beads) and the reporters will be in proximity only when the two receptors form a heterodimer. The VeraTags were released in Monogram’s illumination buffer upon reaction with the molecular scissors beads. Illumination for VeraTag release was performed at room temp for 10 min using Monogram’s 96-array illuminator, which consists of an LED array that can irradiate the plate containing the assay mixture at a wavelength of 680 nm. The released VeraTag molecules were analyzed by CE (ABI 3100). Fluorescein was used as an internal standard for all CE runs to control for injection efficiency. The peaks were identified and the peak areas were calculated as relative peak area (RPA) with an in-house peak quantitation software (VeraTag Informer™) software. The antibodies used in the lysate assays are biotin-labeled HER3 antibodies (1B4C3; Santa Cruz and Ab-7; LabVision), VeraTag-labeled HER2 antibody (Ab-4; LabVision), while the phospho, PI3K and Akt antibodies are the same as those used in the FFPE assay.

##### In the HER3 lysate assay protocol, a filter plate (Millipore MAGVN2250, 0.22 um) was preblocked with 50 µL PBS containing 1 mg/ml BSA. The preblocking solution was filtered off using the Millipore multiscreen vacuum system (pressure set at 5). The filter plate was washed with 50 µL of lysis buffer followed by the addition of 30 µL of sample lysate, 5 µL of biotin labeled 1B4C3 antibody and 5 µL of molecular scissors beads (1 mg/ml in 1 mM TBS, pH 8.0) in a dark room and incubated for 40 min at room temperature on a plate shaker. It is preferable to do a serial dilution of the sample along with control cell lysates. The sample solution was removed from the filter plate as before under vacuum and the plate was washed once with 150 µL of lysis buffer. 40 µL of the VeraTag labeled antibody mix containing the HER2, PI3K, phospho-tyrosine and the second HER3 antibodies were added to each well and incubated for 30 min as before. The plate was washed with 150 µL of PBST followed by two washes with 0.005x PBS. 50 µl of illumination buffer was added and the plate is illuminated for 20 min using Monogram’s 96-array illumination device and placed on shaker for 5 min. The samples (20 µl each) were transferred to an ABI 96-well CE plate and run on ABI 3100 CE instrument on a 22-cm capillary filled with POP-4 separation matrix (ABI) at 15 kV separation voltage for 15 min. Samples were loaded at 6 kV injection voltage for 80 sec. The illumination buffer used contains two separation markers and fluorescein.

*Data analysis*: The VeraTag Informer software provided automated analysis of the VeraTag reporter assay data to determine receptor expression, dimerization and phosphorylation levels. The software is designed to read output files from commercial capillary electrophoresis DNA sequencing instruments like the ABI 3100. Using electrophoretic mobility markers, the VeraTag Informer peak detection and quantitation algorithm automatically detects peaks, identifies the assay-specific VeraTag reporter peaks and quantifies the proteins they represent. The software calculates the area under the curve for each VeraTag peak, and transforms this measurement into Relative Peak Area (RPA) by normalizing the VeraTag peak area to that of the internal standard fluorescein. The RPA is proportional to the initial concentration of the corresponding target.

##### Control Markers: Fluorescein was used as an internal control of injection efficiency and VeraTag recovery in every sample. Illumination reactions were done in the presence of a known amount of fluorescein (typically 2 pM or 0.06 fmole) to control for the recovery of VeraTags. CE Markers 1 and 2 are electrophoretic mobility markers developed by Monogram that can be used to identify the window of separation for the assay-derived VeraTag reporter molecules and fluorescein. The VeraTag Informer software uses the relative time of migration of CE markers 1 and 2 to identify the VeraTag reporter molecules.

***Results:***

The principle of the technology is described in the schematic (Supporting information Figure S1): A pair of antibodies recognizing two distinct epitopes of the same protein or protein complex is labeled with biotin and VeraTag respectively. The antibodies are added to the sample containing their specific target. Subsequently, molecular scissors (streptavidin-conjugated methylene blue abbreviated as SA-MB) are added and binds to the biotin-labeled antibody. Illumination of the reaction results in the light-induced generation of singlet-oxygen and the fluorescent VeraTag reporters in proximity to this reactive species are cleaved from the bound antibody. The VeraTag-containing buffer is then subjected to CE where the released VeraTag molecules are separated and detected. Finally, the data is analyzed by the VeraTag Informer software.

*Antibody characterization and assay optimization.* A series of Monogram-generated and commercially available antibodies for the HER2, HER3, PI3K, phospho-tyrosine and Akt receptors were screened in immunohistochemistry and VeraTag lysate assays to select the most suitable candidates for use in the VeraTag FFPE assays. The antibodies exhibiting high specificity toward their target analyte and high signal-to-background ratio were conjugated separately with both biotin and VeraTag. Each of the antibodies was extensively evaluated for cross-reactivity, sensitivity and non-specific signals. For example, cross-reactivity of the Monogram HER3 antibody B9A11, used in the FFPE assays described was not observed with either HER2 or EGFR (Figure S3).

We chose to develop assays for targets along the HER3/PI3K/Akt pathway as this axis is implicated in a number of tumor types and is an intense focus of drug interventions. MCF7 cells were used for initial assay validation and optimization because this cell line has both the HER2 and HER3 receptors. Consequently, it is susceptible to heregulin (HRG) stimulation but in the absence of the HER3 ligand there is no detectable heterodimer or HER3P or HER3PI3K complex. This ligand-induced fold change of specific heterodimers and phosphoproteins in a growth factor dose-dependant manner can be used as a parameter for assay optimization.

Combinations of HER2- and HER3-specific antibodies were tested in VeraTag FFPE assays on MCF7 cells that were either serum-starved or stimulated with HRG. Generic phospho-tyrosine antibodies were screened for the HER3P assay, in order to avoid issues related to phospho-specific epitope lability. The specificity of the HER3P assay is derived from the use of the HER3-specific antibody. Antibody pairs that generated increase in fluorescence signal in a HRG dose-dependant manner in MCF7 cells were chosen for further optimization. Optimization of the assays followed a standard biochemical assay development workflow that included determining the best antigen retrieval buffer, blocking buffer, wash buffers, titration of antibody concentration and incubation times (data not shown).

*Specificity.* Specificity of antibodies was established by performing isotype control experiments, where the biotin labeled antibody was replaced with the corresponding biotin labeled isotype IgG (Figure S4 A-C). While the background signal obtained from isotype was typically <10%, the assay background signal from NIH3T3 cells was <5% of the specific signal from the positive control. The specificity of the HER3P assay was also demonstrated using an alkaline phosphatase treatment of the tissue section prior to running the assays. The HER3P signal was completely eliminated as a result of the treatment while the immunoreactivity of the non-phosphorylated proteins was largely unaffected (Figure S4 D-F)**.** Upon selecting the HER2, HER3, phospho-tyrosine, PI3K and Akt antibodies, the concentrations of these antibodies were selected via antibody titration experiments that produced the highest signal to background ratios and the greatest fold-changes with HRG stimulation. Competition experiments were also performed to ensure that the antibodies chosen recognized only their targeted analytes (data not shown).

*Assay Performance*. To characterize assay performance parameters such as precision and reproducibility, the assays were performed either in one day with a single operator using eight replicates of each sample, or in four batches with eight replicates of each sample over four days. Figure S5 shows the variation seen for VeraTag assays from precision experiments performed using both stimulated and unstimulated cells. We obtained a dynamic range of 2 logs for the HER23D and the HER3 activation assays (HER3P and HER3PI3K).

For the cell line panel, MDA-MB-231 was chosen as a negative control as it does not express detectable levels of HER3. MCF7 and T47D cell lines were selected for their high HER3 expression, MDA-MB-453 for moderate overexpression of HER2, and SKBR3 for overexpression of HER2. The murine cell line NIH3T3 was used as assay background control for these assays (Table S1). The MCF7, T47D, MB453 and SKBR3 cells were serum-starved overnight and then either used as such or stimulated with 60 nM HRG for 10 min before making blocks (Materials and Methods).
