# Supplementary material for: Profiling the HER3/PI3K Pathway in Breast Tumors Using Proximity-Directed Assays Identifies Correlations between Protein Complexes and Phosphoproteins
Source: PLoS One. 2011 Jan 28;6(1):e16443. doi: 10.1371/journal.pone.0016443 (PMC3030586; doi:10.1371/journal.pone.0016443)
Supplement: Table S1 — Flow cytometric analysis of HER2 and HER3 receptors are reported as number of receptors per cell for all the cell lines used in the study. (DOC) [file pone.0016443.s012.doc]

**Table S1**: Flow cytometric analysis of HER2 and HER3 receptors are reported as number of receptors per cell for all the cell lines used in the study.

| Cell Lines | **HER2 (receptor/cell)** ±**SEM** | **HER3 (receptor/cell)** ±**SEM** |
| --- | --- | --- |
| MDA-MB-231 | 37524±911 | 1869 ±333 |
| MCF7 | 46349 ±1316 | 29584 ±1007 |
| T47-D | 66247 ±784 | 32333 ±672 |
| MDA-MB-453 | 398838 ±5288 | 48116 ±361 |
| MDA-MB-468 | 475 ±87 | 17029 ±61 |
| SKBR3 | 1838707 ±278383 | 37347 ±957 |
| BT474 | 1446696 ±286313 | 31935 ±574 |
| SKOv3 | 795401 ±83129 | 163 ±27 |
| NIH3T3 | 84 | 205 |
| HEK293 Cl1 | 37816 ±967 | 121132 ±4196 |
| HEK293 | 23242 | 1074 |
